# SimRFlow: An R-based workflow for automated high-throughput PBPK simulation with the Simcyp^®^ simulator

**DOI:** 10.3389/fphar.2022.929200

**Published:** 2022-08-25

**Authors:** Hiba Khalidi, Anthonia Onasanwo, Barira Islam, Heeseung Jo, Ciarán Fisher, Rich Aidley, Iain Gardner, Frederic Y. Bois

**Affiliations:** Certara, Simcyp® Division, Sheffield, United Kingdom

**Keywords:** high-throughput, PBPK modelling, simulation, simcyp simulator, R, automated data collection, modular workflow, pharmacokinetics

## Abstract

SimRFlow is a high-throughput physiologically based pharmacokinetic (PBPK) modelling tool which uses Certara’s Simcyp® simulator. The workflow is comprised of three main modules: 1) a Data Collection module for automated curation of physicochemical (from ChEMBL and the Norman Suspect List databases) and experimental data (i.e.: clearance, plasma-protein binding, and blood-to-plasma ratio, from *httk*-R package databases), 2) a Simulation module which activates the Simcyp® simulator and runs Monte Carlo simulations on virtual subjects using the curated data, and 3) a Data Visualisation module for understanding the simulated compound-specific profiles and predictions. SimRFlow has three administration routes (oral, intravenous, dermal) and allows users to change some simulation parameters including the number of subjects, simulation duration, and dosing. Users are only expected to provide a file of the compounds they wish to simulate, and in return the workflow provides summary statistics, concentration-time profiles of various tissue types, and a database file (containing in-depth results) for each simulated compound. This is presented within a guided and easy-to-use R Shiny interface which provides many plotting options for the visualisation of concentration-time profiles, parameter distributions, trends between the different parameters, as well as comparison of predicted parameters across all batch-simulated compounds. The in-built R functions can be assembled in user-customised scripts which allows for the modification of the workflow for different purposes. SimRFlow proves to be a time-efficient tool for simulating a large number of compounds without any manual curation of physicochemical or experimental data necessary to run Simcyp® simulations.

## 1 Introduction

Many initiatives^1^ demonstrate growing interest in shifting preliminary toxicity testing from animals to *in vitro* and *in silico* methods (Wetmore et al., 2015; Bois et al., 2017; Berggren et al., 2017; Moné et al., 2020; Escher et al., 2022). As a result, high throughput testing methodologies are important time-saving and cost-saving approaches that are essential for the characterisation of the kinetics of compounds (Wambaugh et al., 2013; Pearce et al., 2017). The Simcyp® software can perform PBPK simulations that predict internal target exposure of human populations to chemicals or therapeutic drugs, following any type of external exposures (Rostami-Hodjegan and Tucker, 2007; Jamei et al., 2014). Simulations can be performed *ab initio*, and in that case they just require physicochemical information on the chemicals of interest. Simulations can also be supplemented with experimental data relating to a compound’s metabolism and clearance (such as the hepatic intrinsic clearance) as well as information on a compound’s blood and plasma binding (such as blood to plasma ratio, and fraction unbound in human blood plasma).

The manual collection, curation and processing of compound-specific data in preparation for running the simulations is 1) time-consuming (various sources may be needed for collecting the required information) and 2) error-prone (high possibility of mistyping/misquoting values form original data source). Specialist assays may also be required for obtaining *in vitro* blood-binding and clearance-related data to supplement the physicochemical data. In the context of a “manual workflow” using the Simcyp® simulator, physicochemical databases are manually searched for the information necessary for running Simcyp® simulations on the compounds of interest. This is followed by manual data entry either 1) directly into the Simcyp® graphical user interface for each compound at a time, or 2) into Simcyp®’s batch-mode template file (Jamei et al., 2009) which runs all compound simulations sequentially after filling in all manually curated data. In both cases, manual data collection and entry are needed, which contribute to the time-consuming and error-prone aspects of the “manual workflow,” particularly when handling large numbers of compounds.

Following each compound simulation, the Simcyp® simulator returns a large collection of user-specified output plots and tables formatted into a single Excel file containing multiple Excel sheets (Jamei et al., 2013). Despite the thorough nature of the information returned in the Excel sheets, it is not possible to immediately view summary parameters for each simulated compound and compare them with the other simulated compounds. Evidently, this becomes problematic when the Simcyp® outputs are to be used for comparing large numbers of compounds which have an equally large number of Excel files containing numerous Excel sheets. To compare a single plot for all compounds, for example a plasma concentration-time profile, users would have to go through each Excel file and navigate to the specific sheet containing the plasma concentration-time profile plot for all simulated compounds.

The inefficiency of manual data curation as well as the limitations of comparing across Simcyp®’s Excel sheet outputs define a clear need for a framework that is suited for high-throughput purposes. To address this, we propose the highly flexible and efficient SimRFlow (Simcyp®-R workflow) framework which has been specifically designed to automate compound data collection as well as enable a wide range of data visualisation options for easy and clear comparison across simulated compounds. SimRFlow aims to facilitate an intuitive approach for using the Simcyp® simulator for a large number of compounds, and does not require any previous knowledge or training on the simulator. Further, SimRFlow is user-modifiable, where in-built assumptions and decisions can be easily changed to suit different simulation purposes.

We describe the modules available within SimRFlow, starting with the data collection module which uses several databases for automatically collecting physicochemical and experimental pharmacokinetic (PK) data for the compounds of interest. This is followed by the simulation module where version 21 of the human Simcyp® Simulator is called from R and parameterised based on the data from the data collection module. Upon completion of compound-specific simulations, users can view and download tables containing the simulated PK parameters, profiles and predictions. Simultaneously, SimRFlow’s data visualisation module returns a wide range of plots such as simulated concentration-time profiles, relationship plots between different simulated parameters, parameter distribution plots across a population, and cross-compound comparison charts. SimRFlow can be used in two different modes: through a user-friendly R Shiny app (see Supplementary Material), or through making scripts of the user-modifiable R functions (functions will be discussed in this paper).

## 2 Methods

**FIGURE 1:**
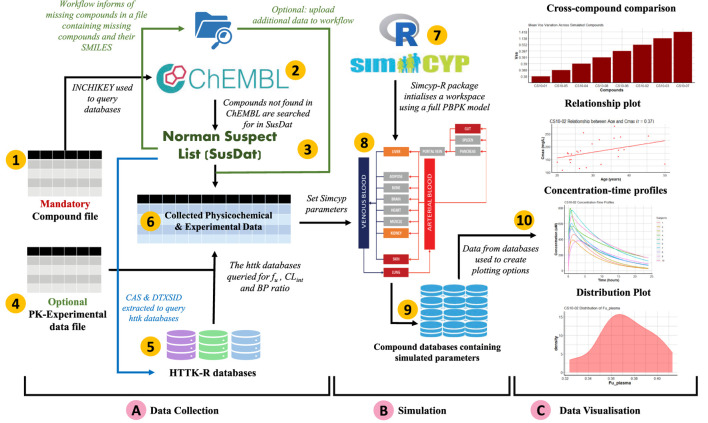
Schematic representation of SimRFlow which is comprised of three main modules as shown by the square brackets **(A)** Data Collection, **(B)** Simulation, and **(C)** Data Visualisation.

Figure 1 shows an enumerated overview of the three modules comprising SimRFlow. Under the data collection module, users are expected to provide a file containing the compounds they wish to simulate (label 1). Physicochemical data available for the listed compounds is firstly extracted from ChEMBL (label 2), and compounds not found in ChEMBL are then searched for in the Norman Suspect List Database- SusDat (label 3). In the instances where users provide a file containing PK-experimental data for the compounds of interest (label 4), the workflow will incorporate this information with the data found in the *httk*-R package databases (label 5). The collected physicochemical and experimental data are pooled together (label 6). Then, the Simcyp® engine is initialised to the human simulator (label 7), and compound-specific simulations are passed through the simulator’s full PBPK model (label 8) using the collected physicochemical and PK-experimental data (label 6). A database file is created for each simulated compound (label 9), and the results from these databases are then used for the data visualisation module of the workflow (label 10).

### 2.1 Physicochemical data collection

#### 2.1.1 Required physicochemical data

The Simcyp® Simulator requires the following physicochemical information on the compounds of interest (Rostami-Hodjegan and Tucker, 2007; Jamei et al., 2014) (See Supplementary Material for parameter definitions):

• Molecular weight (MW)
• *LogP* (also named *LogK*_o:w_)
• The chemical characterisation of the compound as acidic, basic, neutral, or ampholytic
• The logarithm of the acid dissociation constant (*pKa*)
• The polar surface area (*PSA*)
• The number of hydrogen bond donors (*HBD*)

#### 2.1.2 Collecting physicochemical properties

Users must provide a “compound file” (see Section 3.1.1), which is a list of the compounds they wish to simulate and two of their corresponding compound identifiers: The International Chemical Identifier (InChI) key and the simplified molecular-input line-entry system (SMILES) code. InChI keys are used because it is highly improbable that there will be a mismatch between similar compounds when using them to query the databases. SMILES codes are required because they may be used further down the data collection workflow. Before querying the physicochemical databases, the compound file is checked to ensure it contains all the information required to proceed with the workflow. This is done by the ProcessInputs function, which takes the path to the compound file and formats the file into a data structure (processed_cmpndfile) that can be used by subsequent SimRFlow functions (Code Block 1). Users will be notified if the compound file is not properly formatted (see Supplementary Material for further information on formatting the compound file).

Code Block 1 Pre−processing the compound file.

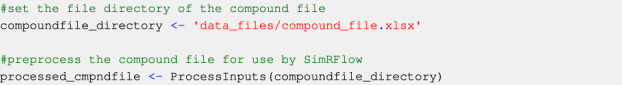

Using the user-provided chemical identifiers, the ChEMBL and SusDat databases are queried as part of the physicochemical data search in SimRFlow (see Table 1 for comparison of the data present in each of the databases used):

1. **The ChEMBL Database:** The user-provided InChI keys are used to query the ChEMBL v29 SQL database (Gaulton et al., 2017) (see section on Data Availability), which contains physicochemical information (see Section 2.1.1) for a large number of compounds. The data extracted from the ChEMBL database are from ChemAxon’s^2^ ChemCurator *in silico* tool. Each compound’s MW, *LogP*, *PSA*, *HBD* count, chemical characterisation, and *pKa* are extracted from ChEMBL v29 if present. Some compounds will have all information available, whilst other compounds might have some physicochemical data missing.
2. **The SusDat Database:** For compounds not found in the ChEMBL database, the open-source Norman Suspect List Database (SusDat) (Network et al., 2022) is queried for MW and *LogP* values, using the user-provided compound InChI keys. SusDat contains both experimental and predicted values of *LogP* taken from the EPI Suite™ database (EPA, 2022). Information collected from the SusDat database is usually sufficient for running a simulation on the Simcyp® Simulator, although additional physicochemical data can improve simulation results.

At the current state of the workflow, ChEMBL must be queried before the SusDat database. The CHEMBLSearch function uses the InChI keys from the processed compound file (processed_cmpndfile) to search for physicochemical data in ChEMBL. The output of the CHEMBLSearch function is a data structure containing all available physicochemical data for the compounds found in ChEMBL (ChemblData). The function NotInChEMBL informs users of the compounds that have not been found in the ChEMBL database (Code Block 2).

**TABLE 1:** Parameters which are extracted from the Physicochemical Databases and *httk* Databases. “(Pred)” refers to predicted values and “(Exp)” refers to experimental values. A checkmark (✓) denotes the presence of the parameter in the database, whilst a cross mark (✗) denotes the absence of the parameter from the database.

	Physchem databases		*httk* databases		
Parameters	ChEMBL	SusDat	Obach 2008	Wambaugh 2019	chem_physical_*invitro*_database
MW	✓	✓	✗	✓	✓
*LogP* (Exp)	✗	✓	✗	✗	✓
*LogP* (Pred)	✓	✓	✗	✓	✓
*pKa* (Pred)	✓	✗	✗	✓	✓
*PSA* (Pred)	✓	✗	✗	✗	✗
*HBD* (Pred)	✓	✗	✗	✗	✗
*LogD* (Pred)	✓	✗	✗	✗	✗
*f*_u_ (*in vitro*)	✗	✗	✓	✓	✓
*CL*_int_ (*in vitro*)	✗	✗	✗	✓	✓
*BP* ratio (*in vitro*)	✗	✗	✗	✗	✓
*CL*_sys_ (*in vitro*)	✗	✗	✓	✗	✗
CAS	✗	✓	✓	✓	✓
DTXSID	✗	✓	✗	✓	✓
SMILES	✓	✓	✗	✓	✓

Code Block 2 Collecting data from ChEMBL and identifying compounds not in ChEMBL.

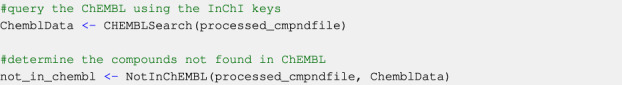

Users may search SusDat for physicochemical data of the compounds which have not been found in CHEMBL (not_in_chembl) using the SusDatSearch function. Any information found in SusDat about the not_in_chembl compounds is appended into the data structure containing physicochemical data from ChEMBL (ChemblData). This returns an updated data structure containing all physicochemical information found in ChEMBL and SusDat (chembl_susdat) for the compounds of interest (Code Block 3).

Code Block 3 Collecting data from SusDat and identifying compounds not in ChEMBL or SusDat.

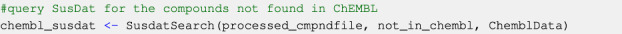

SimRFlow informs users of compounds which have not been found in either of the ChEMBL or SusDat using the MissingInformation function, which returns a data structure of missing compounds (or optionally, information-lacking compounds). If the argument missing_info is set to F, only compounds which have not been found in either of ChEMBL or SusDat will be returned. If the argument missing_info is set to T, compounds which are lacking some physicochemical information (such as *PSA* or *pKa*) will be returned as well as the compounds which have not been found in either of the databases (Code Block 4).

Code Block 4 Identifying missing compounds and compounds with missing information.

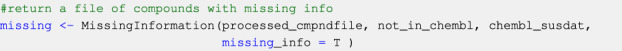

At this stage, users may proceed to the next step of the workflow (Section 2.2) unless they would like to provide additional data to supplement the automatically curated physicochemical data, particularly for the missing compounds. The additional data file (see Section 3.1.3) can include physicochemical information from sources such as ACD/Labs’ Percepta^3^, the S + ADMET predictor^4^, or any other information source or software. The AdditionalData function incorporates the data from the additional data file with chembl_susdat (see the green arrows in Figure 1). Users may choose to either override the automatically curated data by setting the argument override_existing_data = T. This prioritises the information from the additional data file and overrides any overlapping data in the chembl_susdat data structure. If users wish to prioritise the automatically curated data from chembl_susdat over the data in their additional data file, then users must set override_existing_data = F (Code Block 5). Note that searching for and providing this additional data file (for missing or information-lacking compounds) is entirely optional and will not impede the progression of the workflow. However, compounds without any physicochemical data will not be simulated by the Simcyp® simulator.

Code Block 5 Optionally supplementing with additional information.

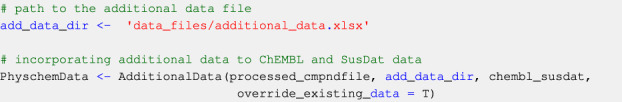

### 2.2 PK-experimental data collection

Compound-specific experimental data may supplement the performance of the simulator. Such data include the fraction unbound in human blood plasma (*f*_u_), the blood-to-plasma (*BP*) ratio, and the hepatic intrinsic clearance (*CL*_int_) - see Supplementary Material for parameter definitions. Users may optionally provide experimental data from custom assays which have been run specifically for the compounds of interest. In the absence of user-provided experimental data, the simulations will proceed using whatever experimental data is available in the *httk* databases.

#### 2.2.1 Databases from the *httk*-R package

Prior to the incorporation of any available user-provided experimental data, three databases from the *httk*-R package (available on CRAN ^5^) (Pearce et al., 2017) are queried for information on *CL*_int_, *f*_u_, and *BP* ratio values in humans. These databases are queried using a combination of compound CAS registry numbers ^6^ and DTXSIDs, ^7^ since querying the databases with only CAS registry numbers might yield unreliable results. CAS and DTXSIDs are collected from SusDat using the function CAS_and_DTXSID (Code Block 6):

Code Block 6 Collecting CAS numbers and DTXSIDs from SusDat.

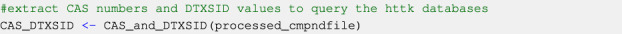

A comparison of data availability across the three *httk* databases can be found in Table 1. The *httk* databases used are:

1. **The “Obach 2008” database:** Compiled from (Obach et al., 2008). The database includes measurements of parameters such as *f*_u_ and systemic clearance (*CL*_*sys*_). At the current state of the workflow, only the extracted *f*_u_ values are used in simulations, while *CL*_*sys*_ values are extracted but not used.
2. **The “Wambaugh 2019” database:** Compiled from (Wambaugh et al., 2019). This database includes measurements of a wide range of human pharmacokinetic parameters. We choose to extract *CL*_int_ and *f*_u_ point-estimates in humans and use those measurements for the simulations.
3. **The “chem.physical_and_invitro.data” database:** is a compilation of measured *in vitro* pharmacokinetic parameters in different species collected from different data sources. We extract only human-related data for *CL*_int_, *f*_u_, and *BP* ratio for the compounds present in this database. Note that this database returns multiple values for *CL*_int_ and *f*_u_ all coming from the same source; therefore, the values are averaged (arithmetic mean) for each compound to provide a single value of *CL*_int_ and *f*_u_ for each compound found in this database.

The httkSearch function uses the collected CAS and DTXSIDs (CAS_DTXSID) to curate the compound-specific PK-experimental data (Code Block 7). The collected data from each of the aforementioned *httk* databases is processed according to user-specified operations (arithmetic mean, geometric mean, median, minimum, or maximum) to provide at most a single value of *CL*_int_ and *f*_u_ for each compound. The data processing for *CL*_int_ and *f*_u_ can be specified through the Clint_operation and fu_operation, respectively. No data processing is applied to the extracted *BP* ratio values since they are only present in the “chem.physical_and_invitro.data” database. Users may introduce new operations to apply them to the extracted *httk* data by editing the underlying code. Alternatively, if users only wish to view the available data and make their own decisions on which data to use, they can set fu_operation and Clint_operation to “None”. However, this returns all values found for *CL*_int_ and *f*_u_ rather than a single value, and users cannot proceed to running simulations before selecting a single value for those parameters. Additionally, users can specify any database combination by setting the database name arguments (obach, wambaugh, and chem_phys_in_vitro) to either T (to include a database) or F (to exclude a database). Users may select only 1 database, any 2 databases, or all 3 databases to be queried at once.

**FIGURE 2:**
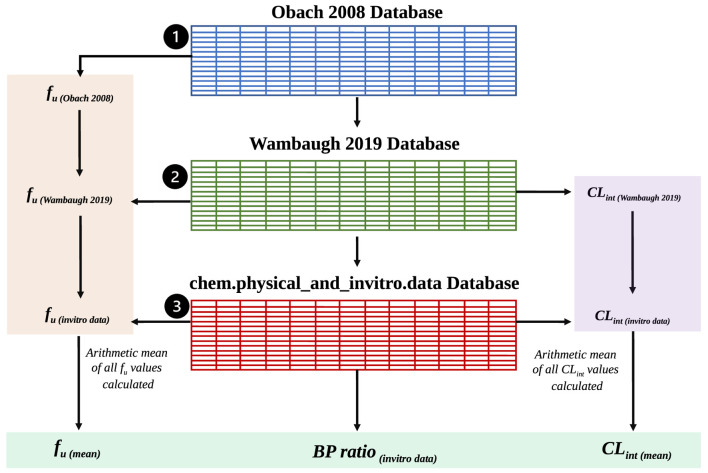
An exemplar data collection and processing workflow where users chose to use the three *httk*-R package databases to extract *BP* ratio and compute the arithmetic means of *CL*_int_ and *f*_u_. Shaded in green are the final parameter outputs from the *httk* databases which will be used for Simcyp^®^ simulations.

Code Block 7 Search the *httk* databases as specified by the exemplar workflow in Figure 2.

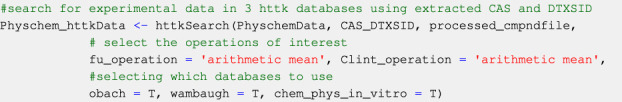

The output of the httkSearch function, Physchem_httkData, is a data structure of all collected physicochemical information as well as the collected PK-experimental data. SimRFlow also computes the standard deviations when there is more than one *CL*_int_ or *f*_u_ value for the same compound present across different databases. The standard deviation is not calculated for *BP* ratio since it is only available in one of the databases. The sources corresponding to the final parameters extracted from *httk* are all noted, and the user is informed of which operation is applied to the *httk* databases to get a single value for *CL*_int_ and *f*_u_. An exemplar workflow of the data collection and processing steps within the *httk* database is visualised in Figure 2.

#### 2.2.2 User-provided measured parameter values

Ideally, users provide measured values for *f*_u_, *CL*_int_, and *BP* ratio for each compound in an experimental data file (see Section 3.1.2). The ExpDataSearch function incorporates any user-provided measured values in their experimental data file to the Physchem_httkData data structure and returns PhyschemExpData which contains compound-specific data curated under the data collection module (Code Block 8). User-provided experimental data is favoured over data from *httk* when the mean_flag is set to FALSE. This means that in the presence of two values for the same parameter for a given compound (from the user-provided experimental data file and from *httk*), the user-provided values will be used. Alternatively, if the mean_flag is set to TRUE, the arithmetic mean of the user-provided values and the *httk* values will be computed instead. It is possible for users to apply thresholds on the values of *f*_*u*_, *CL*_*int*_ and *BP* ratio to be used in the workflow. If not specified, the thresholds for all parameters will be 0 (i.e: all parameter values below 0 will not be used in the simulations).

Code Block 8 Optionally supplementing with user-provided experimental data.

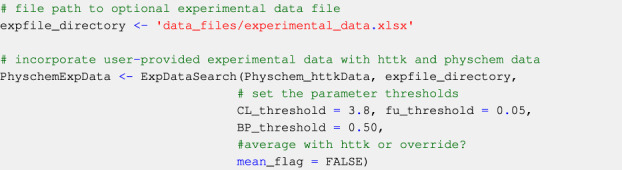

For compounds with unavailable *f*_u_ and *BP* ratio data (not found in *httk* or in the user-provided experimental data file), the Simcyp® simulator predicts the *f*_u_ and *BP* ratio values based on the compound-specific physicochemical information curated (see Section 2.1) (Lobell and Sivarajah, 2003). The Simcyp® predictions will then be used to run the simulations. The assumptions and actions taken for compounds where *CL*_int_ values are missing will be discussed in Section 2.5.

### 2.3 Simulation parameters

To use the Simcyp® simulator as part of SimRFlow, it is necessary to have licensed access to both the Simcyp® simulator and the Simcyp®-R package (both should be version 21). SimRFlow grants users the flexibility to alter simulation design by modifying some simulation parameters. However, other parameters remain fixed and cannot be changed by users at the current state of the workflow.

Code Block 9 Setting some simulation parameters and assumptions.

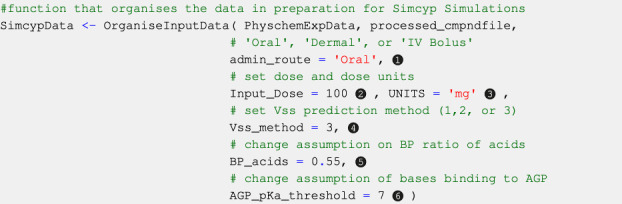

#### 2.3.1 User-modifiable parameters

##### 2.3.1.1 Administration route

Oral, dermal, and intravenous bolus routes of administration are currently available within this version of SimRFlow, and can be changed through the admin_route argument of the OrganiseInputData function (see Code Block 9, label ❶). The bolus intravenous administration is assumed to have an infusion duration of 30 s. The dermal administration route is assumed to be applied to the back with an area of application of 60 cm^2^, a formulation thickness of 0.005 cm, and a formulation density of 1 g/mL. Users can only select one of the three administration routes for each batch of compounds to be simulated. The default administration route is oral, and will be selected if users do not specify a different route of administration. Introducing additional administration routes is underway.

##### 2.3.1.2 Administered dose

Users may choose the dose of compound to be administered to the virtual population by changing the Input_Dose argument of the OrganiseInputData function (see Code Block 9, label ❷). The workflow also allows users to choose different doses for different compounds if the doses have been provided within the mandatory compound file. The default dose value (100 units) will be selected for all compounds if users do not provide a dose value either in the compound file or in the OrganiseInputData function.

##### 2.3.1.3 Dose units

Users may select one of three dose units: milligrams (mg), milligrams per kilogram of body weight (mg/kg), or milligrams per square metre (mg.m^−2^). Similar to the administration dose, users may provide different dose units for the different compounds within the compound file. Alternatively, they may set the same dose units for all compounds through the UNITS argument of the OrganiseInputData function (see Code Block 9, label ❸). The default dose unit is mg. Only single dose administration is allowed as part of SimRFlow, but accommodations for multiple dose scheduling are underway.

##### 2.3.1.4 Steady-state volume of distribution (*V*_ss_) prediction method

The Simcyp® simulator offers three methods for predicting *V*_ss_. Method 1 uses Berezhkovskiy’s modified Poulin and Theil’s method which does not account for the ionisation state of the compound (Berezhkovskiy, 2004; Poulin and Theil, 2009). Method 2 (Rodgers and Rowland, 2007) and Method 3 (Fisher et al. 2019) account for the ionisation state of the compound. However, while Method 2 assumes only unionized fraction can permeate the membranes, Method 3 assumes that a fraction of ionised species can also permeate the membranes depending on the properties of both the compounds and the membranes. SimRFlow allows users to set different *V*_ss_ prediction methods different compounds within the compound file, otherwise, users can specify the *V*_ss_ prediction method of all compound simulations using the Vss_method argument of the OrganiseInputData function (see Code Block 9, label ❹). If not specified, the default prediction method is Method 3.

##### 2.3.1.5 Number of subjects

SimRFlow allows users to specify the number of healthy individuals simulated for the compound simulations (see Section 2.6). This means that all compounds will have their simulations run with the same number of simulated subjects. The number of subjects should be between 1–1,000 individuals. If not specified, the default number of individuals simulated is 10.

##### 2.3.1.6 Simulation duration

SimRFlow allows users to specify the duration of the simulation after dose administration of a compound (see Section 2.6). The minimum duration is 4 h and can be increased by 30 min increments. If the simulation duration is not specified, it defaults to 24 h.

#### 2.3.2 Fixed parameters

The following parameters cannot be changed using SimRFlow unless the Simcyp® workspaces used for the simulations are changed (see Section 2.5):

1. **The Administration Route Parameters:** The Simcyp® simulator allows users to change many administration route parameters such as the duration of intravenous bolus infusion, the area of dermal application, and dosing schedules, all of which are not changeable by SimRFlow’s functions.
2. **The Subject Population:** The Simcyp® simulator supports a wide range of patient populations of different ethnicities, ages, and health conditions. For the time being, only the healthy patient population is used. When running simulations, healthy subjects are randomly selected from a population of individuals between the ages of 20–55 years with an equal likelihood of being male or female.
3. **Effective Permeability (*P*_eff_) Prediction Method:** Currently, SimRFlow only allows for the prediction of *P*_eff_ using compound *PSA* and *HBD* count in the absence of *in vitro* cell-based data (Winiwarter et al., 1998). There are other *P*_eff_ prediction methods which Simcyp® can make use of, those of which may use experimental data from *in vitro* assays.
4. **A First Order Absorption Model:** Two other absorption models are available in Simcyp® under the oral route of administration. These are the “Advanced Dissolution, Absorption and Metabolism Model” (ADAM) and the “multi-layer gut wall within ADAM Model” (M-ADAM). SimRFlow only uses the first order absorption model.
5. **The Single Layer Dermis Model:** For the dermal administration route, the single layer dermis model is only allowed using SimRFlow, whilst Simcyp also offers the options of the depth resolved dermis model and the MechDermA model.
6. **A Full PBPK Model:** Simcyp® offers two modes of simulation: a full PBPK model and a minimal PBPK model. The workflow only utilises the full PBPK model for the time being.

### 2.4 Data processing and assumptions

A primary aim of SimRFlow is to reduce the amount of time taken to make informed decisions and assumptions which are integral to running accurate simulations. A set of assumptions are applied to the collected physicochemical data prior to passing it into the Simcyp® simulator.

#### 2.4.1 Assumptions on compound donation/acceptance of protons

Different data sources provide varying amounts of *pKa*-related information; SusDat provides no compound characterisation or *pKa* values, ChEMBL often states a characterisation for its compounds as well as their predicted *pKa* values, and the user-provided additional data file can contain compound characterisations and a maximum of two *pKa* values. Compounds without a chemical characterisation (from ChEMBL or from the user-provided additional data file) will be classified to acids, bases, neutrals and ampholytes according to their *pKa* values:

• **No *pKa* values:** Compounds without any *pKa* values are assumed to be neutrals. This includes all compound data extracted from SusDat (unless overriden by the user-provided additional data file).
• **One *pKa* value:** Compounds with one *pKa* value are classified as monoprotic acids if their *pKa* value is less than 7, or they are classified as monoprotic bases if their *pKa* value is more than 7.
• **Two *pKa* values:** Compounds with two *pKa* values are classified as diprotic acids if both *pKa* values are more than 7, or they are classified as diprotic bases if both *pKa* values are less than 7, or they are classified as ampholytes if one *pKa* value is more than 7 and the other is less than 7.
• **More than two *pKa* values:** If all *pKa* values for a given compound are greater than 7, the compound is assumed to be a diprotic base, and the two largest *pKa* values are taken. If all *pKa* values for a given compound are less than 7, the compound is assumed to be a diprotic acid and the two lowest *pKa* values are taken. For compounds with *pKa* values both above and below 7, the two *pKa* values farthest away from 7 will be taken, and the compounds will be assumed to be a diprotic acid/base/ampholyte depending on the selected *pKa* values and the 3 rules outlined above.

Once *pKa* values are selected for each compound, they are modified such that they fall within the *pKa* range of 0–14 in order to be accepted by Simcyp®. The *pKa* values which are below 0 are assumed to be 0, whereas *pKa* values above 14 are assumed to be 14. Compounds with no characterisation (acid, base, ampholyte, neutral) and no *pKa* values are assumed to be neutral. Note that the assumptions made on compound donation/acceptance of protons are only exemplar and used in default situation where users do not specify any other assumptions and/or rules for selecting *pKa* values. The *pKa* assumptions applied are tailored to chemical risk assessment, but users may set their own rules to fit the purposes of their research as long as the rules result in a maximum of two *pKa* values for acidic, basic and ampholytic compounds.

#### 2.4.2 *BP* ratio of acids

Acidic compounds lacking information on *BP* ratio (from both *httk* and the user-provided data) are assumed to have a *BP* ratio of 0.55. This assumption is based on the fact that acidic compounds exist in an ionized state in blood (blood pH = 7.4), resulting in an inability to permeate the red blood cells’ lipid bilayer. Since the haematocrit to plasma ratio is 0.45 : 0.55, a conservative estimate of *BP* ratio of acidic compounds would be 0.55, since acids will not be able to permeate into the red blood cells (thereby avoiding the entirety of haematocrit in blood). SimRFlow allows users to make their own assumptions on the *BP* ratio value of acids which do not have an experimental *BP* ratio. This can be done by changing the BP_acids argument of the OrganiseInputData function (see Code Block 9, label ❺) Alternatively, users can abandon this assumption and choose to use Simcyp®-predicted *BP* ratio values.

#### 2.4.3 Plasma protein binding

The Simcyp® simulator offers three plasma protein options for binding of the simulated compound: human serum albumin (*HSA*), alpha (1)-acid glycoprotein (*AGP*), and “other” (which can be specified by the user). SimRFlow decides whether a compound binds to *AGP* or *HSA* based on its physicochemical properties curated in the data collection steps. If a compound is basic with a *pKa* more than 7, it is assumed to bind to *AGP*. Otherwise, all other compounds are assumed to bind to *HSA*.

The plasma protein binding assumption is also exemplar and will be used in default situations where users do not provide a different rule. Users may change the *pKa* threshold for *AGP* binding through the AGP_pKa_threshold argument of the OrganiseInputData function (see Code Block 9, label ❻). Users can also create a new *HSA*/*AGP* binding rule by editing the OrganiseInputData function, or the user can simply manually choose which compounds bind to *AGP* and which to *HSA*. Alternatively, they may wish to abandon this assumption entirely and select either *HSA* or *AGP* binding for all provided compounds.

#### 2.4.4 Calculations of *LogD*_7.4_ and fraction unbound in hepatocytes *f*_u, hep_

For consistency purposes, the value of *LogD* at pH 7.4 extracted from the different data sources is not used in the default scenario (where users do not specify a different rule for this assumption), but instead computed from the compound’s *pKa* using the *LogD*_7.4_ calculation from (Scherrer and Howard, 1977). Using this *LogD*_7.4_ value (whether calculated or extracted from the data sources), the *f*_u, hep_ value is then computed using the Kilford equations (Kilford et al., 2008).

#### 2.4.5 Assumptions on *PSA* and *HBD* count

In cases where data on a compound’s *PSA* and/or *HBD* count is unavailable in all data sources, the workflow assumes the missing values to be 0. Additionally, there are limits on the range of values of *PSA* and *HBD* which the Simcyp® simulator can accept; *PSA* values must be between 0 and 300, and *HBD* count values must be between 0 and 20. Values that are above the limits will be rounded down to the upper limit of the respective parameter.

### 2.5 Activating Simcyp® workspaces through R

To access the Simcyp® simulator software, interested institutions can apply for academic research licenses (email the corresponding authors for enquiries about that). The workflow uses version 21.0.100 of the Simcyp®-R package along with R version 4.1.2 to call the Simcyp® human simulator V21 engine for running compound-specific simulations using Simcyp®’s full PBPK model. Two Simcyp® files (workspaces) are used for each of the three administration routes (6 workspaces in total). The first workspace uses the Simcyp® mechanistic kidney model (MechKiM) and the second workspace does not (Non-MechKiM). Only one of these workspaces is activated for a given compound depending on the availability of *CL*_int_ values:

1. **MechKiM:** This workspace is activated for compounds that do not have any *CL*_int_ information from the *httk* databases or user-provided experimental data file (see section 2.2). Lacking information on hepatic clearance leads to the extremely conservative assumption that the compound of interest cannot be cleared by the liver. It is then assumed that the compound is solely cleared out renally, with a renal clearance equal to the product of *f*_u_ and the glomerular filtration rate (*GFR*). In that case, the most accurate and mechanistic renal excretion sub-model available in Simcyp®, MechKiM, is used (Neuhoff et al., 2013). However, MechKiM runs slower than Non-MechKiM; therefore, more compounds with missing *CL*_int_ values results in longer simulation times.
2. **Non-MechKiM:** The Non-MechKiM workspace is activated for compounds with a known *CL*_int_ value (available either from the *httk* databases) or the user-provided experimental data file. Non-MechKiM considers the liver as the only site of clearance of the compound, and operates under the well-stirred liver model assumption. This assumes that the compound distribution into the liver compartment is perfusion-limited, and that no active transporters are involved in this process (Yang et al., 2007)

### 2.6 Using Simcyp® through R

SimRFlow offers two modules for using Simcyp® through R. The first module is SimRFlow’s prediction module which returns Simcyp® predictions of *f*_u_ (Lobell and Sivarajah, 2003) *BP* ratio, *V*_ss_, and the equilibrium dissociation constant, *K*_d_ (used to calculate *f*_u_) without running any compound-specific simulations. Note that the outputs from the prediction module will not necessarily be used by the workflow, as some compounds might have experimental PK data already available from *httk* or the user-provided experimental data file. The purpose of the prediction module is to provide insight into the nature of the predictions returned by Simcyp® which are based on the compound-specific physicochemical data curated from the data collection module.

The second module offered by SimRFlow is the simulation module. The simulation module runs Simcyp® simulations on a full PBPK model (parameterised using the collected physicochemical and experimental data) following the activation of the appropriate workspace based *CL*_int_ availability. The simulations are run based on the user-specified variables (see section 2.3.1) as well as population size (Code Block 10, label ❼) and simulation duration (Code Block 10, label ❽). Users may also set a seed for the “randomised” subjects in the simulations by setting seed = T (Code Block 10, label ❾) which ensures the same subjects are simulated for each of the compounds. If users wish to have randomised subjects which are different for each compound, they may set seed = F. Running the SimcypSimulation function returns the simulated concentration-time profiles of all simulated subjects of all compounds. Since a full PBPK model is used, the concentration time profiles of 10 tissue types are included: blood plasma, skin, liver, kidney, lung, spleen, brain, heart, gut, and pancreas.

Code Block 10 Running Simcyp simulations.

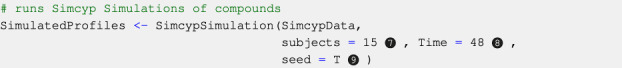

Upon the completion of each compound simulation (executed by the SimcypSimulation function), an SQL database file is created for the simulated compounds titled with the user-provided compound codes. SimRFlow offers functions to extract (AdditionalOutputs, Code Block 11 label ❿) and summarise (SummaryOutputs, label ⓫) additional PK parameters (see Table 2) from the created SQL databases. Users can also create their own R or SQL scripts to extract any additional data from the databases.

**TABLE 2:** The SimRFlow output parameters returned for each compound. These parameters can be referred to in the plotting functions of the data visualisation module.

Output parameters	Units
*AUC*	ng.hr/mL or *μM*.hr
*AUC* infinity	*ng.hr/mL* or *μM* *.hr*
*C*_max_ (all 10 tissue types)	*ng/mL* or *μM*
*T*_max_ (in blood plasma)	*hr*
Half life	*hr*
Absorption rate constant (*k*_a_)	*hr* ^−1^
Predicted *V*_ss_	*L/kg*
Predicted *f*_u_	dimensionless
Predicted *BP* ratio	dimensionless
Fraction absorbed by enterocytes (*f*_a_)^a^	dimensionless
Fraction escaping gut wall metabolism(*f*_g_)^a^	dimensionless
Fraction escaping hepatic metabolism (*f*_h_)^a^	dimensionless
Accumulation index	dimensionless
*GFR*	*ml/min/*1.73 m^2^
Age	Years
Body weight (*BW*)	*Kg*
Body surface area (*BSA*)	*m* ^2^
Total systemic clearance (*CL*_*tot*_)	*L/hr*
Total hepatic intrinsic clearance (*CL*_*H*_)	*L/hr*
Total renal intrinsic clearance (*CL*_*R*_)	*L/hr*

^a^The fraction of the drug at different stages of absorption.

Code Block 11 Extracting additional data from the created SQL files.

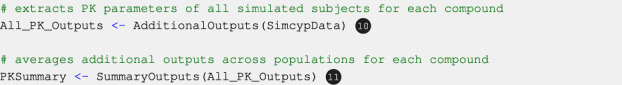

## 3 Results: Running the workflow

SimRFlow requires minimal inputs and provides a wide range of outputs suited for both per-subject and cross-compound comparisons. The workflow is significantly faster than manual data collection. On average, it takes around 2.5 min to manually collect and record data (from any online data source) for a single compound, whereas SimRFlow demonstrated in a test run that it can collect physicochemical and experimental data for 450 compounds in less than 3 s. The display of the workflow is also user-friendly, providing two modes of usage: code-based (by using functions as outlined in the Methods), and app-based (using a R-Shiny app as demonstrated in the Supplementary Material).

### 3.1 Minimal inputs to SimRFlow

SimRFlow requires minimal user input, only requiring a single compound file which contains compound names and their chemical identifiers to run the entire workflow. The design of SimRFlow allows the upload of up to three files for different uses, only one of which is mandatory (see Figure 1 for position of files in the workflow):

#### 3.1.1 The mandatory compound file

This file must either be a CSV (Comma Separated Value) or Excel file containing the following mandatory column headers on the first row of the sheet, spelled exactly as follows (case insensitive): “Code,” “Compound,” “InChIKey,” and “SMILES”. “Code” refers to a user-specified unique code for each compound to be simulated. The codes do not have a specific format and can be made of any combination of letters and numbers. The “Code” is used as a succinct way to refer to a specific compound particularly within the plotting options, where long compound names might occupy multiple lines in plot headers and axes titles. The “Compound” refers to the actual name of the compound, and if no name is provided for the compound, the compound “Code” will be used as replacement. Evidently, the absence of compound names is not detrimental to SimRFlow’s progression, but their presence makes it easier to map which “Code” corresponds to which “Compound”.

The mandatory compound file may also contain any of the optional headers, spelled exactly as follows (case insensitive): “VssMethod,” “Dose,” and “DoseUnits”. “VssMethod” refers to the prediction method of *V*_ss_, which can either be 1, 2, or 3 for Methods 1, 2 and 3, respectively (see Section 2.3.1.4). “Dose” and “DoseUnits” refer to the administered dose value (Section 2.3.1.2) and the units of the dose (Section 2.3.1.3), respectively. Users may specify different doses, dose units, and methods of *V*_ss_ prediction for the different compounds within the mandatory compound file. If these optional headers are not passed as part of the mandatory compound file, users may select the dose, dose units, and *V*_ss_ prediction method through the OrganiseInputData function or from the SimRFlow application interface (see Supplementary Material for SimRFlow’s RShiny app). If users do not select specific value for these parameters either through the functions of through the app, the default parameters will be used (see Table 3).

**TABLE 3:** The user-modifiable parameters and their defaults. Defaults are applied when users do not specify values for these parameters.

Parameter(s)	User-inputs	Default parameters
Administration Route	Can be “Oral,” “Dermal,” or “Intravenous Bolus”	Oral
Dose	Any value >0 can be specified	100
Dose Units	Can be either *mg*, *mg/kg*, or *mg.m* ^−2^	*mg*
Method of *V*_ss_ Prediction	Can be either 1, 2 or 3	3
Number of Individuals	Any value between 0 and 1,000	10 individuals
Simulation Duration	Any value >4 hours in increments of 30 min	24 h

The absence of any of the mandatory column headers means that the workflow cannot proceed to the next steps. Although column headers are mandatory, entries under the column headers are optional (*i*.*e*.: It is possible to have a compound file with some compounds missing SMILES, InChI keys or compound names. A missing InChI keys means that the compounds will not be simulated). Both the mandatory and optional headers can appear in any order, and may be separated by any number of columns. Non-mandatory/non-optional headers and any entries below them will be ignored. See example of acceptable compound files in the Supplementary Material.

#### 3.1.2 The optional PK-experimental data file

In case where users provide experimental data, the file must be an Excel file with the mandatory column headers, spelled as follows (case insensitive): “Code,” “FU,” “BP” and “CLINT,” corresponding to the compound code (see Section 3.1.1), *f*_u_, *BP* ratio, and *CL*_int_, respectively. SimRFlow will be capable of handling missing entries under the mandatory headers appropriately by either activating the correct workspace for missing *CL*_int_ (Section 2.5), or predicting values of missing *f*_u_ and *BP* ratio. The optional experimental data file may contain any number of compounds which have been provided in the mandatory compound file, each of which does not necessarily need to have entries for *f*_u_, *BP* ratio, and *CL*_int_. The absence of any of the mandatory column headers means that the workflow cannot incorporate the user-provided experimental data. See examples of acceptable experimental data files in the Supplementary Material.

#### 3.1.3 The optional additional physicochemical data file

In cases where users can provide additional compound-specific physicochemical data, they may upload the additional data file directly into SimRFlow. The additional physicochemical data file must be an Excel file containing only one mandatory header, spelled as exactly as follows (case insensitive): “Code”. Users can optionally include additional headers containing physicochemical data, but in order for the entries to be recognised by SimRFlow, the headers must be spelled as follows (case insensitive): “logP,” “pka(acid),” “pka(base),” “HBD,” “PSA,” “MW,” and “TYPE”. Where “pka(acid),” “pka(base)” and “TYPE” correspond to the acidic *pKa*, basic *pKa* and the compound characterisation, respectively. See examples of acceptable additional physicochemical data files in the Supplementary Material.

### 3.2 A diverse set of outputs

Using only the mandatory compound file, SimRFlow returns many downloadable tables at several steps of the “Data Collection,” “Prediction” and “Simulation” modules. Following the completion of all compound simulations, users have access any of the four plotting tools specifically designed for the visualisation of the highly dimensional simulated outputs. Each plotting tool comes with optional parameters which can be used for aesthetic customisation, and selection of *x* and *y* axes specifications.

#### 3.2.1 Data tables

Users may view and download tables of the collected data at various steps within the “Data Collection” module. This provides insight into the hierarchy of the data curation process, as well as an understanding of the logic being applied in the background of the workflow. SimRFlow also returns tables containing simulated concentration-time profiles and predicted parameters that could be of interest to the users. All data tables provided by the workflow are downloadable in CSV and Excel formats, except when specified otherwise.

##### 3.2.1.1 Data collection module tables

The following tables are returned as part of the data collection module:

1. **Processed Compound File:** Upon upload of the compound file, users can immediately see which columns have been extracted for use in the workflow. The processed compound file will have at least the four mandatory headers (“Code,” “Compound,” “InChIKey,” and “SMILES”), and any/all of the three optional headers, if present (“VssMethod,” “Dose,” and “DoseUnits”). This table is not downloadable.
2. **Physicochemical Data:** Following the curation of physicochemical data from ChEMBL and SusDat, users may view the table containing the curated data. This table contains information on MW, *LogP*, *pKa*, compound characterisation, *PSA*, *HBD*, and the sources of the collected information.
3. **Missing Compounds:** Simultaneous to the creation of the physicochemical data table, users can see another table containing compounds (with their SMILES) which have not been found in either ChEMBL or SusDat. This table can be downloaded as a CSV file for use in further data collection (*i.e:* for the creation of the additional data file).
4. **Physiochemical Data with Additional Data (Optional):** Users may upload additional physicochemical data into SimRFlow. The data is automatically incorporated into the physicochemical data table, and can be immediately viewed and downloaded. In cases where additional physicochemical data is unavailable, this data table will not be produced.
5. **PK-Experimental Data:** Following the search for PK-experimental data in *httk* and the user-provided experimental data file (if provided), users can view a table containing all curated physicochemical and experimental data organised in a single downloadable table.

##### 3.2.1.2 Prediction and simulation module tables

The following tables are returned as part of the prediction and simulation modules:

1. **Prediction Module Table:** The outputs form SimRFlow’s prediction module is a table containing compound-specific predictions of *f*_u_, *BP* ratio, *V*_ss_, and *K*_d_ values in *HSA* and *AGP.*
2. **Simulated Concentration-Time Profiles:** After simulations for all compounds are completed, SimRFlow returns a large table containing all the compounds’ simulated concentration-time profiles for all simulated subjects in various tissue-types. Concentration-time profiles for blood plasma, brain, skin, liver, kidney, lung, spleen, heart, gut and pancreas are all returned.
3. **Simulated Summary Data:** A summary of the simulated compound-specific predictions along with their standard deviations across the simulated populations are returned. For example, summary predictions include the mean “maximum plasma concentration” (*C*_max_), time for *C*_max_ (*T*_max_) and their corresponding standard deviations within the simulated population. The summary data table will contain the parameters listed in Table 2 for all simulated compounds.

#### 3.2.2 Plotting capabilities

##### 3.2.2.1 concentration-time profiles

Concentration-time profile plots are created with concentration on the *y*-axis and time (in hours) on the *x*-axis. Users can view concentration-time profiles in both logarithmic and natural scaling of the *y*-axis with concentration units of either *μM* or *ng/mL* as specified by the user. The concentration-time profile plot contains a legend of subject identifiers which correspond to the same subjects in the concentration-time profile table (see Section 3.2.1.2). The profiles of up to 30 subjects can be plotted for each simulated compound. In cases where more than 30 subjects per compound are simulated, the profiles of 30 randomly chosen subjects will be plotted in order to avoid over-crowding and preserve plot clarity. Users can select any of the 10 available tissue types to create the concentration-time profiles.

##### 3.2.2.2 Relationship plots

Relationship plots are scatter plots where users can select any two parameters from Table 2 to be plotted against each other across all simulated subjects for a selected compound. Relationship plots allow the user to select which variable to be plotted on each of the *x* and *y* axes. The plot is displayed with a header that includes a correlation coefficient (*r*) which indicates the strength of the relationship between the two user-selected variables. A trend line (line of best fit) is fitted into the scatter plot using a linear model.

##### 3.2.2.3 Parameter distribution plots

For each simulated compound, the distribution of the simulated parameters across the population of simulated subjects can be viewed on density plots. Density plots provide a visual understanding of the most probable value that a simulated parameter can take and the range of values which this simulated parameter occupies. Users can select different colours for their density plots and choose to view the distributions of any of the simulated parameters in Table 2.

##### 3.2.2.4 Cross-compound comparison charts

As part of the high-throughput cross-compound comparison, the workflow provides an option for visualising the different predicted parameters averaged across all simulated subjects for each compound. User-selected parameters (from Table 2) are plotted in bar charts across all simulated compounds, and users may choose to display the bar charts either in alphanumeric order of the compound codes, or in ascending order of the predicted parameter values.

### 3.3 Example of using SimRFlow

**FIGURE 3:**
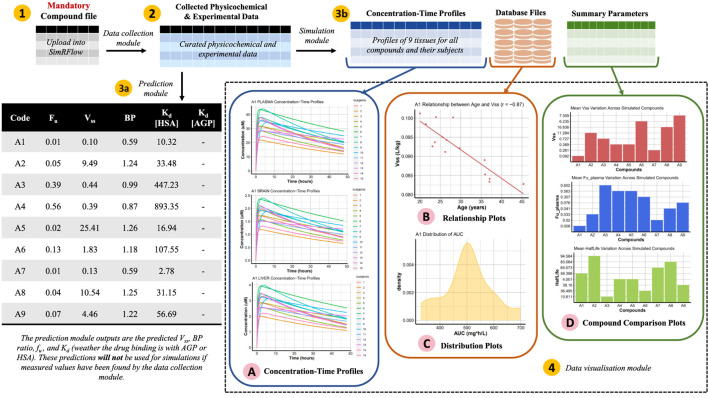
Schematic representation of SimRFlow’s functions being applied on the exemplar compound file in Table 4 (label 1). The data collection module generates a table of the curated physicochemical and experimental data (label 2) which it uses to activate the prediction module (label 3a) or the simulation module (label 3b). The data visualisation module returns a range of plotting options for understanding the simulation outputs (label 4).

SimRFlow’s method of operation and capabilities are demonstrated in this section using nine compounds of varying levels of data availability. The compound file is uploaded to the workflow (Figure 3, label 1) and the data collection module is activated (Figure 3, label 2). At this point, it is possible to activate either the prediction module (Figure 3, label 3a) or the simulation module (Figure 3, label 3b). If the simulation module is used, the simulated outputs are used to generate the plots under the data visualisation module (Figure 3, label 4). This section will discuss this exemplar workflow in detail and demonstrate the output results.

#### 3.3.1 Creating the compound file

For this example, we choose nine compounds with varying levels of data availability to test SimRFlow’s performance. These compounds are formatted into the mandatory compound file with their corresponding InChI keys and arbitrary compound codes (Table 4). SMILES are not necessary for data collection and so they have not been included as part of the compound file in this example (although the SMILES header is still present). Compounds A2, A5, A6 and A8 have been categorised as having high data availability, where high data availability compounds are those which have all physicochemical data and a maximum of one missing value from either *f*_*u*_ or *BP* ratio. Compounds A1, A3, A7 and A9 have been categorised as having intermediate levels of data availability, where intermediate data availability compounds are those with all physicochemical data and at most two missing experimental data values (*f*_*u*_, *BP* ratio, or *CL*_*int*_). Finally compound A4 is categorised as having poor data availability, where poor data availability refers to having limited physicochemical data and no experimental data. The compound file (Table 4) is uploaded into SimRFlow and passed into the ProcessInputs function (Code Block 1) which ensures that the compound file is properly formatted.

**TABLE 4:** An exemplar compound file of nine compounds containing all compound InChI keys. Codes are arbitrarily generated for the compounds. The SMILES header is present but has no entries beneath it; this will not impede the progression of the workflow as only InChI keys are required for automated data collection.

Code	Compound	InChIkey	SMILES
A1	Fluvastatin	FJLGEFLZQAZZCD-MCBHFWOFSA-N	
A2	Midazolam	DDLIGBOFAVUZHB-UHFFFAOYSA-N	
A3	Colchicine	IAKHMKGGTNLKSZ-INIZCTEOSA-N	
A4	Butoxyacetic Acid	AJQOASGWDCBKCJ-UHFFFAOYSA-N	
A5	Dibutyl Phthalate	DOIRQSBPFJWKBE-UHFFFAOYSA-N	
A6	Lidocane	NNJVILVZKWQKPM-UHFFFAOYSA-N	
A7	Atrovastatin	XUKUURHRXDUEBC-KAYWLYCHSA-N	
A8	Bisphenol A	IISBACLAFKSPIT-UHFFFAOYSA-N	
A9	Bisphenol F	PXKLMJQFEQBVLD-UHFFFAOYSA-N	

#### 3.3.2 Using the data collection module

SimRFlow uses the InChI keys of the processed compound file (processed_cmpndfile) (Figure 3, label 1) to activate the data collection module:

1. **Physicochemical Data Collection from ChEMBL:** Physicochemical (*MW*, *LogP*, *PSA*, *HBD*, LogD, *pKa*) and SMILES data from ChEMBL are collected using the CHEMBLSearch function. To check which compounds have not been found in ChEMBL, the NotInChEMBL function is used (Code Block 2).
2. **Physicochemical Data Collection from SusDat:** Physicochemical (*MW* and *LogP*) and SMILES data of the compounds not found in ChEMBL are searched for in SusDat using the SusdatSearch function (Code Block 3). To check which compounds have not been found in either in ChEMBL or SusDat, the MissingInformation function is used (Code Block 4). See the physicochemical data collected for the nine compounds of interest in the Supplementary Material.
3. **No Additional Physicochemical Data:** In this example, we do not provide an additional physicochemical data file to incorporate with the data found in ChEMBL and SusDat. Therefore, the physicochemical data (chembl_susdat) to be used for simulations will only be whatever data has been collected from ChEMBL and SusDat (Code Block 3).
4. **PK-Experimental Data Collection from *httk*:** CAS and DTXSID values (CAS_DTXSID) are collected from SusDat using the CAS_and_DTXSID function (Code Block 6). In this example, we choose to use all 3 *httk* databases and apply an “arithmetic mean” operation on the collected *f*_u_ data and a “minimum” operation on the collected *CL*_int_ data (Code Block 12). Note that the default of the httkSearch function is to use all three databases, so there is no need to pass the database names as arguments to the function. See the experimental data collected for the nine compounds of interest in the Supplementary Material.

Code Block 12 Exemplar search of the *httk* databases.

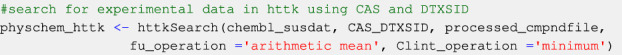

5. **No User-Provided Experimental Data:** In this example, we do not provide any additional experimental data for the compounds of interest. This means that any experimental data collected from the httkSearch function will be used for running the Simulations (Code Block 12).

This concludes the data collection module where physicochemical and experimental data for our nine compounds have been collected. See the Supplementary Material for the full tables of collected physicochemical and experimental data.

#### 3.3.3 Organising the collected data

To use the simulation and prediction modules, the collected data is organised into a format which can be easily processed by Simcyp®. The organisation of the data involves setting some trial design parameters (⓬) and assumptions (⓭). In this example, acid *BP* ratio (for acids without an experimental *BP* ratio) is set to 0.55, and the *pKa* threshold for binding to *AGP* is set to 7.5 (⓭). The simulations will have a single dose of 25 *mg* administered orally for all compounds, and the prediction method for *V*_*ss*_ is set to Method 3 (⓬).

Code Block 13 Organising Simcyp® data.

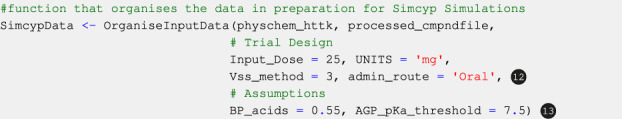

#### 3.3.4 Using the prediction module

Code Block 14 Using the prediction module.

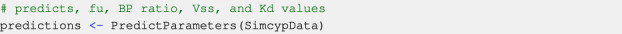

In this example, we use the prediction module (Figure 3, label 3a) before using the simulation module. Users do not need to activate the prediction or simulation modules in order to access the other; they can use whichever module in their preferred order. The prediction module uses the organised data (SimcypData) to return Simcyp®’s predictions of *f*_*u*_, *BP* ratio, *V*_*ss*_ and *K*_*d*_ based on the compound-specific physicochemical data collected in Section 3.3.2. The prediction module ignores the collected experimental data (*i.e: f*_*u*_ and *BP* ratio values from *httk*) and the trial design parameters (Code Block 13 ⓬) which are only used as part of the simulation module. The PredictParameters function (Code Block 14) returns Simcyp®’s predictions which are shown in the table in Figure 3.

#### 3.3.5 Using the simulation module

After using the prediction module, we choose to use the simulation module (Figure 3, label 3b). The simulations are run on 15 healthy volunteer subjects for 48 h, and a seed is set to ensure that all simulated subjects are identical for all compounds (see Code Block 10). The output of the SimcypSimulation function are database files containing the simulated profiles and parameters for each compound (see “Database Files” in Figure 3). The concentration-time profiles in all 10 tissue types for all subjects in all compounds are extracted from the database files (see “Concentration-Time Profiles” in Figure 3). All additional data is extracted and summarised using the AdditionalOutputs and SummaryOutputs functions (Code Block 11), respectively.

#### 3.3.6 Using the data visualisation module

The data visualisation module (Figure 3, label 4) relies on the data from the simulation module to create the four plot types of the “plottable parameters” (Table 2). Compound codes from the mandatory compound file must be used to refer to a compound when using the plotting options. Three of the nine simulated compounds have been chosen based on their data availability to demonstrate the different plotting options. Compound A2 is a data-rich compound, where all of its physicochemical properties and PK-experimental data have been found by the data collection module. Compound A4 is a data-poor compound which does not have any PK-experimental data and only its *MW* and *LogP* values have been found by the data collection module. Compound A9 has moderate data availability; its physicochemical properties and measured *CL*_*int*_ values have been found by the data collection module, but its *f*_*u*_ and *BP* ratio values are missing.

##### 3.3.6.1 Concentration-time profiles

Code Block 15 Function to plot concentration-time profiles.

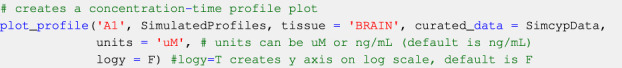

**FIGURE 4:**
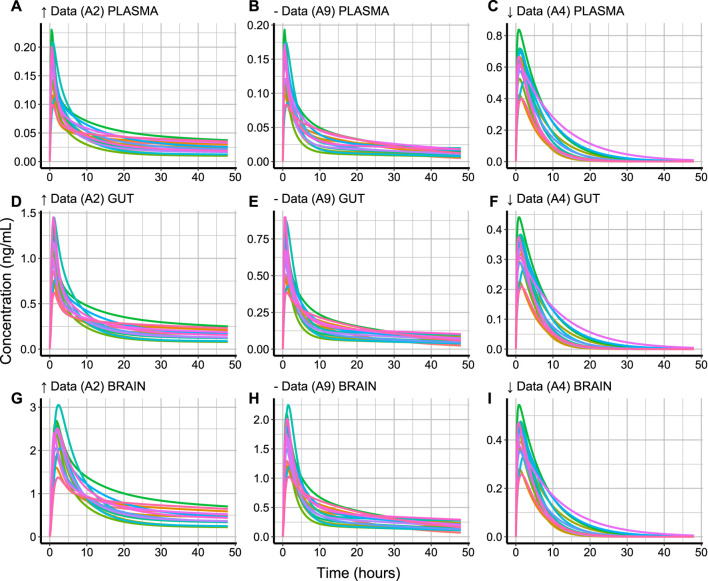
Simulated concentration-time profiles (*ng/mL*) in 15 healthy subjects for 48 h in **(A–C)** plasma, **(D–F)** gut and **(G–I)** brain for compounds with high (*↑* Data, A2), medium (− Data, A9), and low (*↓* Data, A4) data availability. The different subject profiles are colour-coded.

To plot the concentration-time profile of a compound, the compound code and tissue type must be specified in the plot_profile function. It is also possible to select the concentration units and the *y* axis scaling when displaying the plots as shown in Code Block 15. The concentration-time profiles of the three compounds with varying levels of data availability are plotted as shown in Figure 4.

The variation of a compound’s concentration appears to have a very similar profile in the different tissue types. However, different tissue types reach different *C*_*max*_ values for the same compound. The rate of elimination of compound A4 (Figures 4C,F,I) from the plotted tissue types appears to be slower than that of compounds A2 (Figures 4A,D,G) and A9 (Figures 4B,E,H). This can be explained by the lacking *CL*_*int*_ value for A4, which caused SimRFlow to activate the MechKiM. Compounds A9 and A2 have a higher *C*_*max*_ in brain tissue (Figures 4G,H) when compared with their *C*_*max*_ in blood plasma or gut tissue. This is different for A4, where its highest *C*_*max*_ value is in blood plasma (Figure 4C).

##### 3.3.6.2 Relationship plots

Code Block 16 Function to create a relationship plot.

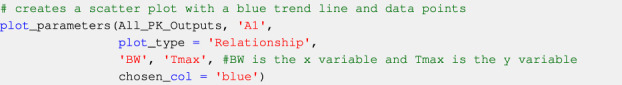

**FIGURE 5:**
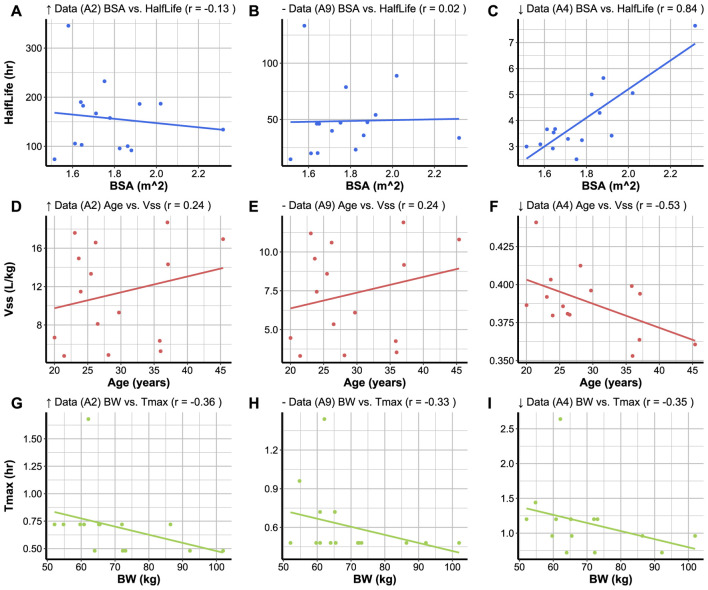
User-chosen variables for *x* and *y* axes are plotted against each other with the correlation coefficient (*r*) in the plot headers. **(A)**, **(D)**, and **(G)** display the relationships between variables for a compound (A2) with high data availability (*↑* Data). **(B)**, **(E)**, and **(H)** display the relationships between variables for a compound (A9) with medium data availability (− Data). **(C)**, **(F)** and **(I)** display the relationships between variables for a compound (A4) with low data availability (*↓* Data). The plots **(A–I)** have been created based on 15 subjects randomised with the same seed for each simulation.

To create a relationship plot between two parameters simulated for a compound, the compound code must be specified, and the plot_type must be set to “Relationship” in the plot_parameters function (Code Block 16). The first variable to be specified is the *x* variable and the second to be specified is the *y* variable. It is also possible to select the colour of the data points and trend line using the chosen_col argument. The relationship plots of the three compounds with varying levels of data availability are plotted as shown in Figure 5.

The relationship between *T*_*max*_ and BW (Figures 5G–I) is consistent across the simulated compounds despite the varying levels of data availability the relationship between; where an increase in BW shows a trend in decreasing the *T*_*max*_. The *r* value between *T*_*max*_ and BW is very similar across the simulated compounds. There appears to be no relationship between the strength of *r* and the amount of data availability for a given compound. Compound A4 (with low data availability, Figure 5C) demonstrates a stronger relationship between half life and BSA than that simulated for compounds A2 and A9 (with high and medium data availability, Figures 5A,B, respectively). In other cases, different compounds may demonstrate opposite relationships between the same parameters; for example, compounds A2 and A9 demonstrate a weak positive relationship between *V*_*ss*_ and age (Figures 5D,E), whilst compound A4 demonstrates a moderate negative relationship (Figure 5F).

##### 3.3.6.3 Distribution plots

Code Block 17 Function to create a distribution plot.

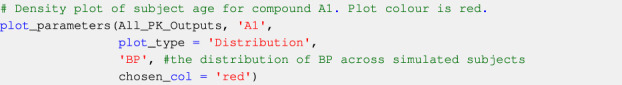

**FIGURE 6:**
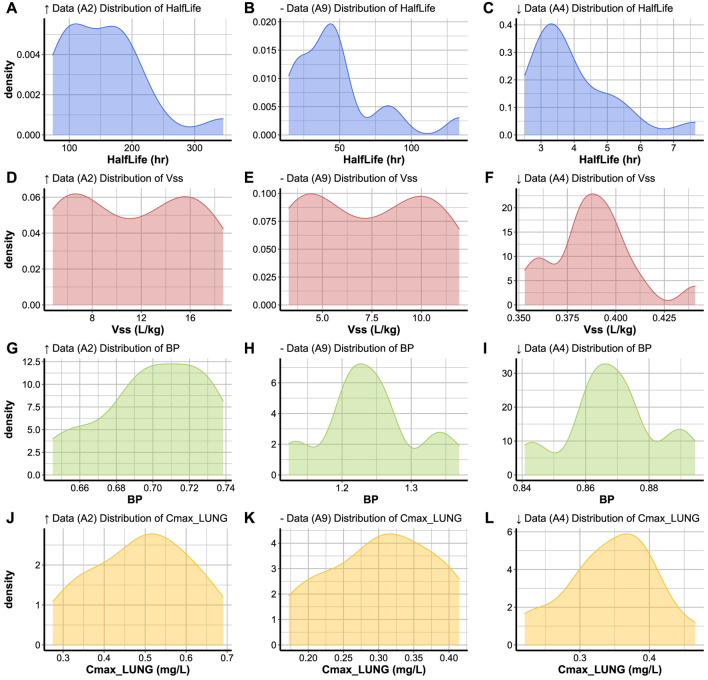
Variation of **(A–C)** Half life, **(D–F)** *V*_*ss*_, **(G–I)** *BP* ratio, and **(J–L)** *C*_*max*_ in lung within the same population of 15 subjects simulated for compounds with high (*↑* Data), medium (− Data) and low (*↓* Data) data availability.

To visualise the distribution of a parameter simulated for a set of individuals for a certain compound, the plot_type is set to ’Distribution’ in the plot_parameters function (Code Block 17). The parameter to be plotted is the only parameter to be specified when activating the distribution function. The distribution plots of the three compounds with varying levels of data availability are shown in Figure 6. The distribution of a parameters across a population of individuals sometimes covers a large range of values (i.e: half life distribution for compounds A2 and A9, Figures 6A,B) or a narrow range of values (i.e: half life distribution for A4, Figure 6C). Simulated parameters for a population are sometimes normally distributed around the parameter mean (such as *C*_*max*_ in lung values, Figures 6J,L), or appear to have a positively skewed normal distribution (such as the half life distribution in A4, Figure 6C). The distributions can sometimes appear to resemble bimodal distributions (such as the *V*_*ss*_ distribution, Figures 6D–F), or multimodal distributions (such as the *BP* ratio distribution for compounds A9 and A4, Figures 6H,I).

##### 3.3.6.4 Cross-compound comparison charts

Code Block 18 Function to create cross-compound comparison charts.

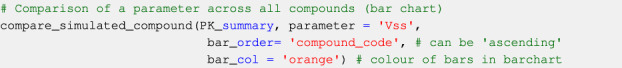

**FIGURE 7:**
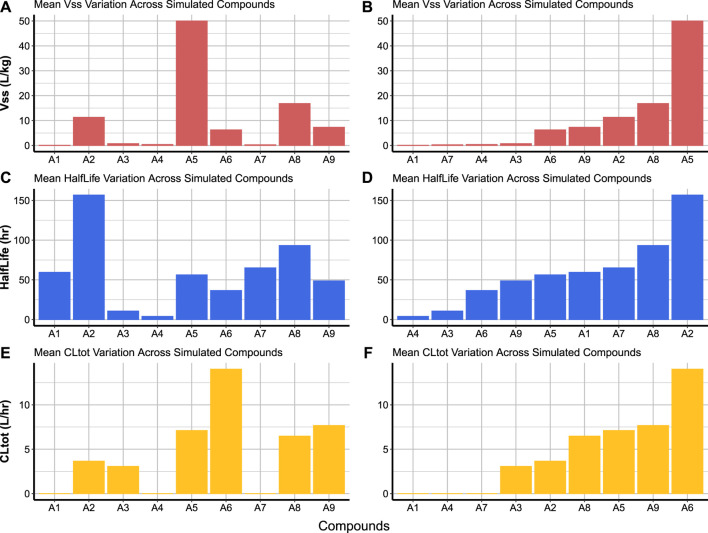
Cross-compound comparison charts allow the visualisation of different parameter means for each compound across the simulated populations. The bars can be organised in the alphanumeric order of compound codes as in panels **(A)**, **(C)**, and **(E)**, or in ascending order of parameter values as in panels **(B)**, **(D)**, and **(F)**.

Cross-compound comparison charts are used to compare the average of a parameter (over the simulated subjects) across the simulated compounds. This is done by passing the created summary of Simcyp® simulations (PK_summary) to the compare_simulated_compound function (Code Block 18). It is possible to compare any parameter from Table 2 and also indicate whether compounds are displayed in alphanumeric order of compound codes (Figures 7A,C,E) or in ascending order of parameter value (Figures 7B,D,F). Compounds A1, A4 and A7 are the only compounds which did not have experimental *CL*_*int*_ data. The lack of experimental *CL*_*int*_ made SimRFlow activate the MechKiM workspace, contributing to a very conservative total systemic clearance (*CL*_*tot*_) prediction for these compounds as opposed to the other compounds (Figure 7E, F).

## 4 Discussions

### 4.1 Comparison with similar tools

Multiple tools are capable of executing a workflow similar to that of SimRFlow. Perhaps the most similar tool is the *httk*-R package which is developed by the United States Environmental Protection Agency. *httk*-R contains several databases of physicochemical and experimental parameters for 553 chemicals (both predicted and measured), as well as tissue data that is used for partition coefficient calculation in humans, rats, mice and rabbits. The following models are available in *httk*: a dynamic physiologically-based toxicokinetic (PBTK) model, a 3-compartment model, and a 1-compartment model. These can be used for the prediction of chemical concentrations in different tissues through oral or intravenous administration routes in user-specified population demographics. The package also offers some plotting options and tools that can conduct *in vitro* to *in vivo* extrapolation (Pearce et al., 2017).

Tebby et al.’s PBPK model in Euromix’s MCRA (Monte Carlo Risk Assessment) Platform can predict compartment-specific kinetics for nine compounds administered dermally, orally or through inhalation. The models are parameterised using measured *in vivo* and *in vitro* data for the nine compounds in humans and rats. The expected accuracy of the model’s are based on the different ‘data availability’ conditions, and users are informed of the extent of accuracy of the predictions. ‘Data availability’ refers to the presence of 1) compound structural data, 2) *in vivo* data, and 3) *in vitro* data. Different combinations of the different data types yield different expected model accuracies. It is often the case that greater data availability for a specific compound yields better prediction of PBPK compartment kinetics (Tebby et al., 2020).

A tool tailored for chemical safety assessment is the SEURAT “*ab initio*” workflow which is capable of predicting the point of departure and margin of safety for a given chemical with a quantified uncertainty associated with the predictions. The SEURAT “*ab initio*” workflow adopts a hierarchical approach with increasing confidence levels for estimating a dose which is safe under repeated administration (Berggren et al., 2017).

Simcyp® has been tested on many compounds and is mechanistically more sophisticated than models under *httk* and the MCRA Platform. The Simcyp® simulator is also capable of handling varying levels of data availability; this allows detailed compound kinetics to be simulated in cases where only *MW* and *LogP* are present. Further, SimRFlow’s data visualisation and plotting options can provide clarity in distinguishing between the different compounds/parameters as well as inter-subject variability for hundreds-thousands of compounds. Table 5 summarises the differences between the similar workflows offered under the aforementioned tools.

**TABLE 5:** Comparison of SimRFlow performance with that of *httk*, the MCRA Platform and SEURAT *ab initio* workflows.

	*httk*	MCRA platform	SEURAT *ab initio*	SimRFlow
**Limit on number of compounds**	553	9	NA	No limit
	compounds	compounds		
**Populations**	Many populations	Healthy	Healthy	Healthy
	(user-specified	human	human	human^a^
	demographics)			
**Species**	Humans, rats	Humans, rats	Humans	Humans^a^
	mice, rabbits			
**Administration Routes**	Oral, intravenous	Dermal, oral	Dermal, oral	Dermal, oral
		inhalation		intravenous
**Handling data availability**	Can handle	Can handle	Unable to handle	Can handle
	high and low	high and low	low data availability	high and low
	data availability	data availability	scenarios	data availability
**Prediction accuracy**	No	Yes	Yes	No

**Publicly available**	Yes	No	No	Yes

**Suitable for**	Yes	No	No	Yes
**high-throughput**				
**Manual data**	No	Yes	Yes	No
**curation required**				
**User-modifiable**	Yes	No	No	Yes

a Additional human and animal (rat, mouse, dog) populations will be introduced in the next version of the workflow.

### 4.2 Evaluation of SimRFlow

#### 4.2.1 Strengths

SimRFlow is a time-efficient, high-throughput framework for running Simcyp® simulations within an R-based code framework for a large number of compounds (see Supplementary Material for a demonstration of SimRFlow’s high-throughput capabilities). SimRFlow is robust, scalable and able to efficiently handle small and large compound batches (from tens of compounds to thousands), especially within the data collection module of the workflow which has minimal computational complexity. Obviously, the speed and efficiency of the simulation module is entirely dependant on the user’s device’s computational power as well as the number of simulated subjects and specified simulation duration.

In addition, SimRFlow is very user-friendly and easy to use. For users with knowledge of R, the in-built R functions of SimRFlow are publicly available and downloaded from a dedicated GitHub page (https://github.com/mba16hk/Simcyp-R-Workflow). The scripts are accompanied with a detailed instructions manual as part of the Supplementary Data, and each line of script is commented at every decision step. With full understanding of the scripts’ capabilities and limitations, users may edit or modify the in-built assumptions/decisions to adjust them for different purposes. This makes the workflow very flexible to user needs and requirements.

SimRFlow offers a graphical interface through an R Shiny application which contains separate pages for each of the modules. The first page corresponds to the Data Collection module (see Sections 2.1–2.2), the second page is for Parameter Prediction (see Section 3.2.1.2), the third page is for Running Simulations (see Section 2.5), the fourth page is for Data Visualisation (see Sections 3.2.2.1–3.2.2.4), and a final section contains a help file. Users are guided from the moment the application is opened through pop-up messages which appear upon hovering over the different elements of the app’s interface. Users can jump between the different modules, but can only access certain capabilities of the workflow when other steps have been completed (for example, the ‘Simulate’ button will not appear in the app if the data collection steps have not been completed).

SimRFlow’s output tables can be downloaded and used for further data analysis or custom plotting. Alternatively, the data can be used upstream of another separate user-designed workflow. The database files created for each compound are also readily accessible within the user-specified directory, and contain more detailed data (enzymatic and pathway profiles, population demographic data, transporter turnover, tissue-specific clearance values, *… etc*.) than that returned by SimRFlow tables. The additional data can then be extracted from the database files for further analysis. It is also possible to see the Simcyp®-based predictions of *f*_u_, *BP* ratio, *V*_ss_, and *K*_d_ values in *HSA* and *AGP* in bulk, (and maybe compare them with available experimental data) without running any simulations using SimRFlow’s prediction module.

Despite its high-throughput nature, SimRFlow returns a wide range of outputs in human-readable format. Most importantly, the four available plotting options are easily accessible for all simulated compounds and provide a clear way to compare simulation results, not only across subjects for a given compound, but also across all compounds simulated. All of the plotting options are available within a single dialog box, and users can select the different compounds or PK parameters which they wish to view.

#### 4.2.2 Limitations

Multiple trade-offs are associated with the usage of high-throughput Simcyp® simulations compared to the direct usage of the Simulator for running compound-by-compound simulations. SimRFlow restricts the users ability to change and select from a wide range of options which are readily editable within the Simulator’s interface, and only allows for some editable parameters (see Section 2.3.1). Conversely, Simcyp® allows users to select certain enzyme interactions, specify clearances in different organs, set dosing schedules, change population demographics (age, ethnicity, health and pregnancy status, male to female ratio, *… etc*.), all of which are out of reach within SimRFlow. Further, the high-throughput nature of the workflow constrains the reporting of simulation outputs to only the key results in order to keep them human-readable (but the remaining outputs are stored in the results database files, as mentioned previously).

Further, conservative predictions may arise because of the conservative assumptions applied (outlined in Section 2.4). Although this allows for more cautious decisions, it might not necessarily be representative of the real effects of a compound, particularly if many assumptions are made due to missing experimental data. For example, a *BP* ratio value of 0.55 leads to conservative estimates of the concentration of an acidic compound in blood (see Section 2.4.2). Similarly, using MechKiM when *CL*_int_ values are missing implies an extreme assumption that a compound cannot be cleared by the liver, resulting in significantly lower simulated clearance and metabolism (as with the case of compounds A4 in Figure 4 and Figure 7).

There are limitations associated with the use of the *P*_eff_ prediction model which solely relies on *PSA* and *HBD* values. An understanding of the assumptions of the model used in the workflow is essential since it was built under the assumption that compounds with a 16.2 ≤ *PSA* ≤154.4 will have 0 ≤ *HBD* ≤5 , and 60 ≤ *MW* ≤455 (Winiwarter et al., 1998). Compounds with properties that do not fall within the limits of this *P*_eff_ prediction model will have uncertainty associated with their simulations. This can be addressed by using different *P*_eff_ prediction models which will be incorporated in future versions of the workflow (see Section 4.3).

A final limitation of the workflow is that the *CL*_int_ values obtained from the datasets within *httk* may not necessarily be within the linear range of the *in vitro* assays. Substrate depletion assays for estimating *CL*_int_ should be performed well below the Michaelis constant (*i.e.*, under linear conditions). The *CL*_int_ values that lie outside the upper and lower bounds of the *in vitro* assays are not accurate and should not be used for kinetic estimation. The compounds with *CL*_int_ values outside the lower or higher bound of the *in vitro* assays should be estimated with alternate systems suitable to estimate lower or higher *CL*_int_ values.

### 4.3 Improvements and future work

Upcoming versions of SimRFlow will offer different patient populations (different diseases, ethnicities, and age groups), allow the setting of age ranges, male-to-female ratios, and dosing schedules for the different compounds, and have an inhalation administration route. A continued effort will be invested in the improvement and expansion of the available plotting options. Additional options such as plot shape, and size will be introduced, and all plots will be downloadable in a range of formats. Additional data visualisation options, such as heatmaps, will also be introduced to better understand the variation of all simulated parameters for the simulated compounds.

Further, current limitations of the workflow will be addressed. Users will be given the option of opting out of using *httk* data, or only using Simcyp®’s predictions of experimental parameters (without using *httk* or user-provided data). An additional functionality will be introduced to allow the selection of which physicochemical databases to rely on, and if more than one is chosen, users will be able to specify an order for querying the different databases based on their preferences and priorities. In order to address the limitations associated with the use of the *PSA*/*HBD P*_eff_ prediction model, we hope to allow users to upload data from various *in vitro* assays within their experimental data file. This would then be processed by the workflow and the most accurate *P*_eff_ prediction method would be selected accordingly.

As an extension of the functionality of SimRFlow, the integration of additional modules for reverse dosimetry will be a priority. Users will be able to use data extracted from the simulations to carry out estimations of the toxicity threshold concentrations of different compounds. Alternatively, pharmacokinetic equations can be used alongside SimRFlow’s prediction module to conduct the reverse dosimetry calculations and arrive at a point of departure that is not dissimilar to that calculated from the simulated data.

## 5 Conclusion

SimRFlow is a high-throughput workflow which uses the Simcyp® full PBPK model to simulate profiles and predict parameters for many compounds at a time. The workflow initiates this process by looking for compound-specific physicochemical and experimental data within free-access databases. Physicochemical data is collected from ChEMBL then SusDat (and other data sources, if available), which is then followed by experimental data collection for some relevant PK parameters from three *httk* databases. In cases where users have experimental data relevant to their compounds of interest, they may also upload it as part of the workflow. Evidently, the specifics of the data collection and processing steps can be modified and customised by users to fit their purposes. SimRFlow then applies a set of decisions and assumptions regarding the physicochemical and experimental parameters and processes the data in preparation for compound simulations in the Simcyp® simulator. These decisions and assumptions can also be changed by users. Where experimental data is lacking, the workflow addresses this by appropriately activating the experimental parameters prediction modules in Simcyp®, and the predicted parameters are then used to run the simulations. Each compound is then simulated sequentially, and a database file containing all simulated parameters and profiles is produced for each compound and stored within a user-specified directory. The workflow returns a plethora of data including compound and subject-specific concentration-time profiles, summary outputs of the predicted parameters for the simulated population, and offers four plotting tools designed to compare simulated outputs across different compounds and within the simulated populations. The design of this end-to-end efficient framework ensures that there are no human errors in pulling the data from the original data source and into the simulator. Further, the data visualisation options allow for a quick and reliable way to compare the predicted pharmacokinetic behaviour of different compounds on the same population of individuals.

## Data Availability

The source code of SimRFlow can be found on this public GitHub repository: https://github.com/mba16hk/Simcyp-R-Workflow. The ChEMBL v29 SQL Database can be downloaded from https://ftp.ebi.ac.uk/pub/ 927 databases/chembl/ChEMBLdb/releases/chembl_29/. The edited Norman Suspect List Dataset (SusDat) can be downloaded directly from the GitHub repository (SusDat is provided under a Creative Commons License) at: https://github.com/mba16hk/Simcyp-R-Workflow/blob/main/data_files/Norman_susdat.zip. The data_files/ directory contains an exemplar compound file, experimental data file and an additional data file containing dummy data. The link to the most recent versions of ChEMBL and SusDat databases will be updated annually on the GitHub page, and the scripts will be updated to accommodate the most recent versions of the databases.

## Footnotes

1 See https://www.risk-hunt3r.eu/, https://www.eu-toxrisk.eu/, https://www.nc3rs.org.uk/, https://www.euromixproject.eu/

2
https://chemaxon.com/

3
https://www.acdlabs.com/

4
https://www.simulations-plus.com/

5
https://cran.r-project.org/web/packages/httr/index.html

6
https://www.cas.org/

7 DTXSIDs are DSSTox substance identifiers used by the United States Environmental Protection Agency CompTox dashboard https://comptox.epa.gov/dashboard/downloads
