# Supplementary material for: SimRFlow: An R-based workflow for automated high-throughput PBPK simulation with the Simcyp® simulator
Source: Front Pharmacol. 2022 Aug 25;13:929200. doi: 10.3389/fphar.2022.929200 (PMC9455594; doi:10.3389/fphar.2022.929200)
Supplement: Supplementary file 1 [file DataSheet1.PDF]

# Supplementary Material

## 1 PARAMETER DEFINITIONS

### 1.1 Physicochemical Properties

- *LogP* (also named $LogK_{\mathrm{o:w}}$) and *LogD*: The logarithm of the octanol to water partition coefficient (*LogP*), which is a measure of lipophilicity of the compound. The partition coefficient is the ratio of the concentration of the solute between water and a non-polar solvent (octanol). It accounts for only the unionised solute. *LogD* is the distribution coefficient which takes into account the ionised and unionised solutes.
- *pKa* is the negative logarithm of the acid dissociation constant (*Ka*), which measures the extent of dissociation of a compound into an acid.
- The polar surface area (*PSA*; $\AA^2$): A quantification of the molecular surface area associated to oxygen, nitrogen, phosphorus, and polar hydrogen atoms.

### 1.2 PK-Experimental Properties

- **Fraction unbound in human blood plasma ($f_u$):** which is regarded as the proportion of the concentration of a compound not bound to plasma proteins to the total concentration of a compound in blood. The extent of binding depends on the extent of affinity the compound has to plasma proteins Watanabe et al. (2018). The $f_u$ value can range from 0 to 1.
- **Blood/Plasma (BP) ratio:** refers to the ratio of the total concentration of a compound in blood to the total concentration of the compound in plasma Hinderling (1997).
- **Hepatic Intrinsic Clearance ($CL_{int}$):** refers to the intrinsic ability of the liver to metabolise a drug and consequently result in its elimination from the body Chiba et al. (2009).

## 2 FORMATTING INPUT FILES

This section provides examples on the 3 different input files and what they can look like. Please follow the guidance in the main manuscript when creating and formatting the input files.

| COMPOUND | Form | SMILES | INCHIKEY | CAS | CODE | MW | DOSE | MF |
|---|---|---|---|---|---|---|---|---|
| n-butyltin trichloride | Liquid | CCCC[Sn](Cl)(Cl)Cl | YMLFYGFCXGNERH-UHFFFAO | 1118-46-3 | COMP1 | 282.18 | 130 | C4H9Cl3Sn1 |
| dibutyltin dichloride | Solid | CCCC[Sn](Cl)(Cl)CC CC | RJGHQTVXGKYATR-UHFFFAO | 683-18-1 | COMP2 | 303.85 | 120 | C8H18Cl2Sn1 |
| tributytin chloride | L | CCCC[Sn](Cl)(CCCC )CCCC | GCTFWCDSFPMHHS-UHFFFA | 1461-22-9 | COMP3 | 325.51 | 110 | C12H27Cl1Sn1 |
| 4-methylimidazole | S | Cc1c[nH]cn1 | XLSZMDLNRCVEIJ-UHFFFAOY | 822-36-6 | COMP4 | 82.11 | 100 | C4H6N2 |
| butanone oxime | L | CC/C(C)=N/O | WHIVNJATOVLWBW-SNAWJC | 96-29-7 | COMP5 | 87.12 | 90 | C4H9O1N1 |
| ethylene thiourea | S | S=C1NCCN1 | PDQAZBWRQCGBEV-UHFFFA | 96-45-7 | COMP6 | 102.16 | 80 | C3H6N2S1 |
| 2-mercaptobenzimidazole | S | Sc1nc2ccccc2[nH]1 | YHMYGUUIMTVXNW-UHFFFAC | 583-39-1 | COMP7 | 150.2 | 70 | C7H6N2S1 |
| 2-methylimidazole | S | Cc1ncc[nH]1 | LXBGSDVWAMZHDD-UHFFFA | 693-98-1 | COMP8 | 82.11 | 60 | C4H6N2 |

Figure S1: The mandatory compound file must have the 4 mandatory headers and may have any of the 3 optional headers on the first line of the Excel/CSV sheet. The workflow recognises the headers of interest (highlighted in amber) and use the entries below these headers in the next steps of the workflow. The workflow is case insensitive; for example, ‘SMILES’, ‘Smiles’ and ‘smiles’ will all be recognised.

| CODE | MW | FU | BP | CLINT |
|---|---|---|---|---|
| COMP1 | 113.16 | 0.6 | 1.1 | |
| COMP2 | 89.09 | 0.92 | 0.8 | 16.3 |
| COMP3 | 102.09 | 0.33 | | 72.5 |
| COMP4 | 127.57 | | 0.55 | |
| COMP5 | 133.19 | 0.01 | | |
| COMP6 | 165.63 | | 0.76 | 11.1 |
| COMP7 | 150.17 | | | 61.3 |

Figure S2: The optional experimental data file must have the 4 mandatory headers on the first line of the Excel/CSV sheet. The workflow recognises the mandatory headers (highlighted in amber) and uses the available entries in the workflow. Users do not need to have have experimental data for all compounds, and may also provide compounds with only some experimental data

| Code | LogP | pKa(Acid) | HBD | PSA | MW |
|---|---|---|---|---|---|
| COMP1 | 0.8 | | 0 | 0 | 282.18 |
| COMP2 | 1.51 | | 0 | 0 | 303.84 |
| COMP3 | 3.69 | | 0 | 0 | 325.51 |
| COMP4 | 4.48 | | 4 | 55.28 | 299.54 |

Figure S3: The additional physicochemical data file must have the ‘Code’ header and any optional headers on the first line of an Excel sheet. The workflow recognises the mandatory and optional headers and uses the available entries to supplement the physicochemical data sourced from ChEMBL and SusDat. Note that SimRFlow is case insensitive, but all headers must be spelled exactly as listed above (with no additional spaces, separators, or characters).

# 3 OUTPUTS FROM EXAMPLE IN MANUSCRIPT

**Table S1.** Physicochemical data collected for the 9 compounds from ChEMBL v29 and SusDat (contains EPI Suite data). The data in this table is the raw, unprocessed physicochemical data. Further down the workflow, this physicochemical data will be processed; for example, the *pKa* values of neutral compounds will be ignored, and compounds without a 'Type' are assumed to be neutral.

| **Code** | **MW** | **LogP** | **LogD** | **Type** | ***pKa1*** | ***pKa2*** | ***PSA*** | ***HBD*** | **Source** |
|---|---|---|---|---|---|---|---|---|---|
| A1 | 411.47 | 3.83 | 1.05 | ACID | 4.54 | - | 82.69 | 3 | ChEMBL v29 |
| A2 | 325.77 | 3.97 | 3.95 | NEUTRAL | - | 6.19 | 30.18 | 0 | ChEMBL v29 |
| A3 | 399.44 | 1.46 | 1.46 | NEUTRAL | - | - | 83.09 | 1 | ChEMBL v29 |
| A4 | 132.08 | 0.79 | - | - | - | - | - | - | EPI SUITE (EPA) Estimated Data |
| A5 | 278.35 | 4.63 | 4.63 | - | - | - | 52.6 | 0 | ChEMBL v29 |
| A6 | 234.34 | 2.84 | 2.33 | NEUTRAL | 13.78 | 7.75 | 32.34 | 1 | ChEMBL v29 |
| A7 | 558.65 | 5.39 | 2.43 | ACID | 4.31 | - | 111.79 | 4 | ChEMBL v29 |
| A8 | 228.29 | 4.04 | 4.04 | NEUTRAL | 9.78 | - | 40.46 | 2 | ChEMBL v29 |
| A9 | 200.24 | 3.46 | 3.46 | NEUTRAL | 9.84 | - | 40.46 | 2 | ChEMBL v29 |

**Table S2.** Experimental data collected for the 9 compounds from the 3 *httk* databases. At the current state of the workflow, systemic clearance ($CL_{sys}$) values are not used for simulations.

| Code | $f_u$ ± SD | $f_u$ source | $CL_{sys}$ | $CL_{sys}$ source | BP | BP source | $CL_{int}$ ± SD | $CL_{int}$ source |
|---|---|---|---|---|---|---|---|---|
| A1 | 0.01 | mean of Obach 2008, Lombardo 2018 | 16 | Obach 2008 | - | - | - | - |
| A2 | 0.03 ± 0.02 | mean of Obach 2008, Wambaugh 2019 | 5.3 | Obach 2008 | 0.67 | Sternbeck 2012 | 22.1 ± 18.8 | Wambaugh 2019 |
| A3 | 0.61 | mean of Obach 2008, Lombardo 2018 | 2.1 | Obach 2008 | - | - | 0.7 | Tonnelier 2012 |
| A4 | - | - | - | - | - | - | - | - |
| A5 | 0.03 | Tonnelier 2012 | - | - | - | - | 42.5 | Tonnelier 2012 |
| A6 | 0.31 ± 0.02 | mean of Obach 2008, Shibata 2002 | 16 | Obach 2008 | 0.84 | Shibata 2002 | 5.1 | Wood 2017 |
| A7 | 0.02 | Wood 2017 | - | - | - | - | - | - |
| A8 | 0.04 ± 0.01 | Wambaugh 2019 | - | - | 0.79 | TNO | 19.7 ± 6.7 | Wambaugh 2019 |
| A9 | - | Wambaugh 2019 | - | - | - | - | 20.5 ± 8.1 | Wambaugh 2019 |

**Table S3.** Running Simcyp® simulations for 50 compounds to demonstrate SimRFlow's high-throughput nature. The data collection and simulation took no more than 7 minutes for the 50 compound to be simulated at a single dose of 25 *mg* for 15 hours and 3 subjects. All predicted parameters and profiles were generated for the 50 compounds, but we demonstrate only the $T_{max}$ and $C_{max}$ values.

| Code | $T_{max}$ (hr) | $C_{max}$ (ng/mL) | Code | $T_{max}$ (hr) | $C_{max}$ (ng/mL) |
|---|---|---|---|---|---|
| **B1** | 0.68 | 0.075 | **B26** | 0.77 | 0.02 |
| **B2** | 1.00 | 1.872 | **B27** | 0.45 | 0.263 |
| **B3** | 0.59 | 0.145 | **B28** | 0.67 | 0.065 |
| **B4** | 1.56 | 0.591 | **B29** | 0.72 | 0.093 |
| **B5** | 1.85 | 2.421 | **B30** | 0.63 | 0.149 |
| **B6** | 0.59 | 0.069 | **B31** | 0.63 | 0.162 |
| **B7** | 0.34 | 0.042 | **B32** | 1.01 | 0.574 |
| **B8** | 0.57 | 0.111 | **B33** | 0.63 | 0.536 |
| **B9** | 0.38 | 0.099 | **B34** | 0.67 | 0.191 |
| **B10** | 0.72 | 0.053 | **B35** | 0.64 | 0.169 |
| **B11** | 0.71 | 0.461 | **B36** | 2.16 | 2.571 |
| **B12** | 0.44 | 0.243 | **B37** | 2.80 | 2.469 |
| **B13** | 1.63 | 1.272 | **B38** | 1.76 | 2.714 |
| **B14** | 0.63 | 0.156 | **B39** | 2.45 | 0.003 |
| **B15** | 1.99 | 2.665 | **B40** | 0.67 | 0.191 |
| **B16** | 1.05 | 1.398 | **B41** | 0.90 | 0.091 |
| **B17** | 1.01 | 1.297 | **B42** | 1.46 | 2.138 |
| **B18** | 1.12 | 0.585 | **B43** | 1.18 | 1.657 |
| **B19** | 4.94 | 0.001 | **B44** | 0.73 | 0.246 |
| **B20** | 0.91 | 0.328 | **B45** | 0.88 | 0.096 |
| **B21** | 0.49 | 0.234 | **B46** | 1.61 | 2.389 |
| **B22** | 0.63 | 0.093 | **B47** | 5.39 | 2.937 |
| **B23** | 0.63 | 0.15 | **B48** | 0.67 | 0.19 |
| **B24** | 0.63 | 0.531 | **B49** | 0.61 | 0.135 |
| **B25** | 0.50 | 0.144 | **B50** | 0.73 | 0.246 |

# 4 PRELIMINARY STEPS TO USING SIMRFLOW

The steps must be completed before using SimRFlow. Failure to complete step 5 will mean that users cannot use the simulation and plotting modules of SimRFlow. Note that these steps should only be done prior to using SimRFlow for the first time.

1. Download the contents of SimRFlow's GitHub page `https://github.com/mba16hk/Simcyp-R-Workflow`.
2. Download the ChEMBL SQL v29 Database from `https://ftp.ebi.ac.uk/pub/databases/chembl/ChEMBLdb/releases/chembl_29/`.
3. Unzip the ChEMBL database and place it inside the downloaded data_files/chembl_29_sqlite directory.
4. Ensure you have R version 4.1.2, and install the following packages from the CRAN repository: httk, dplyr, RSQLite, XLConnect, stringr, tools, tidyverse, Shiny, Shinydashboard, shinydashboardPlus, DT, shinyBS, and shinyWidgets.
5. Install Simcyp® v21 human simulator as well as a zipped file of the Simcyp®-R package (email corresponding author for academic access). The zipped Simcyp®-R package must be installed into R.

# 5 SIMRFLOW R FUNCTIONS

## 5.1 Function Import

To use all functions, users must pre-run all scripts containing the different functions of the workflow:

```
source('input_query.R') # pre-process mandatory compound file
source('chembl_search.R') # search the chembl database
source('susdat_search.R') # search the SusDat database
source('Additional_data.R') #handles additional physicochemical data
source('ACD_Labs.R') #handles ACD/Labs inputs/outputs
source('httk_search.R') #search the httk R library
source('experimental_data_search.R') # incorporate user experimental data
source('organise_simulation_data.R') # prepare inputs for Simcyp
source('R Workflow.R') # simulate compounds in bulk using Simcyp
source('PredictParams.R') # predict parameters through Simcyp
source('plotting_functions.R') # contains all plotting functions
```

## 5.2 ACD/Labs Data

Users who have access to ACD/Labs Percepta software may use the software to find additional physicochemical data. The outpts from the `MissingInformation` function can be used to create a specially formatted CSV file of missing compounds (and/or compounds with missing physicochemical data) for immediate import into ACD/Labs Percepta (containing compound Codes and SMILES). The `ACD_outputs` function is used for incorporating the additional data from ACD/Labs Percepta to that which has been automatically curated by the data collection module. Similar to the additional data file, only values not available in the original physicochemical file will be appended from ACD/Labs into the rest of the physicochemical data.

```
#create a CSV file of missing compounds for import to ACD/Labs
acd_data <- MissingInformation(data, nf_in_chembl, sus_data, missing_info=T)

######## ----------------------------- #########
# 1. user uploads acd_data into ACD/Labs Percepta
# 2. user saves the outputs from Percepta as xls file
######## ----------------------------- #########

# file path to outputs downloaded from ACD/Labs
ACD_data_directory<- 'data_files/acd_output.xls'

# incporates ACD/Labs data with the rest of the physchem data
physchem_data <- ACD_outputs(data, ACD_data_directory, sus_data)
```

# 6 SIMRFLOW R SHINY APP: EXAMPLE OF USAGE

## 6.1 Front Page

As soon as the R Shiny app of SimRFlow is opened, users will see the front page (Figure S4) which allows them to navigate to all other pages from the sidebar menu.

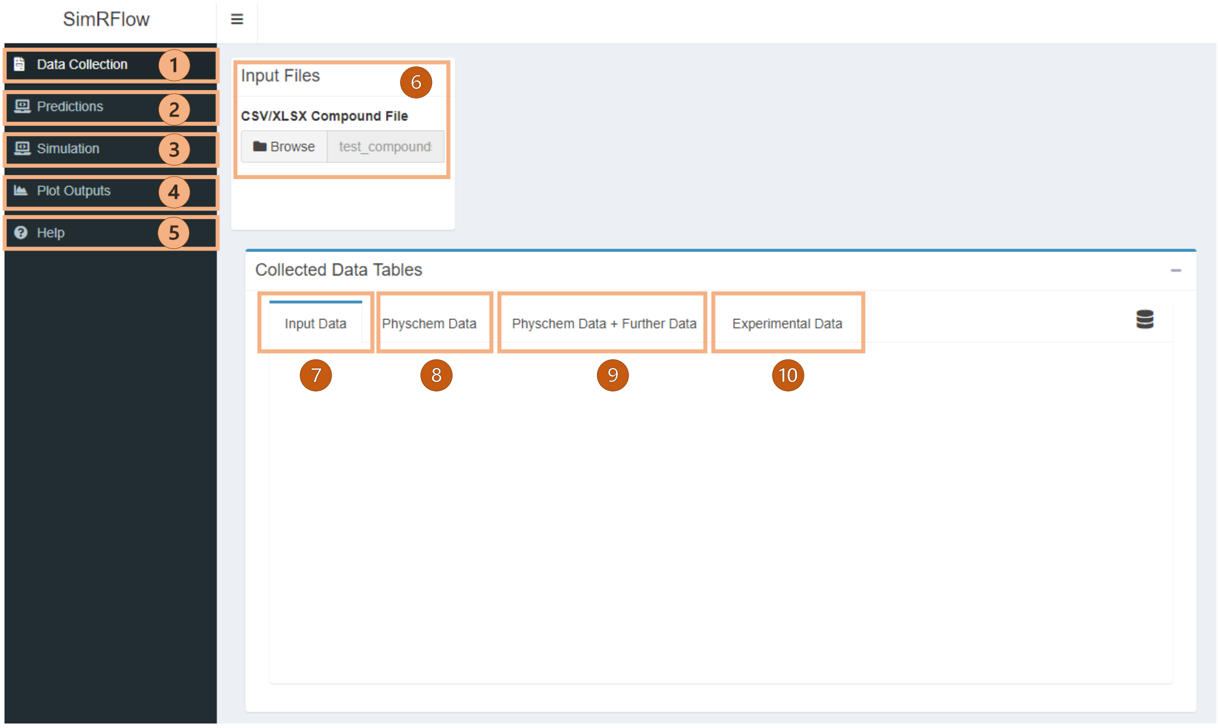


Figure S4: The front page of SimRFlow's R Shiny app. The side panel allows for navigation to different modules of the workflow, and the main panel contains the users working environment where they will be providing inputs and seeing outputs.

The orange labels in Figure S4 correspond to the different elements of the front page. Label 1 is lit up, indicating that the user is currently in the 'Data Collection' module of the workflow app. Clicking on any other sidebar menu item will move the user to that page. Note that the Prediction (label 2), Simulation (label 3), and Data Visualisation (label 4) modules are not usable without completing all necessary steps in the Data collection (label 1) module. The final item in the side bar menu is 'Help' (label 5), which contains some basic guidelines on using the R Shiny app.

In order to begin using the Data Collection module, users are prompted to input the mandatory compound file under the **CSV/XLSX Compound File** in the 'Input Files' box (label 6). Users may click on the browse button to navigate to their mandatory compound file and upload it into the workflow. Upon upload of the mandatory compound file, users will immediately see their processed file in the 'Input Data' tab under the 'Collected Data Tables' box (label 7). As users proceed into the data collection module sub-steps, additional tables will appear under Physchem Data (label 8, for the collected physicochemical data from ChEMBL and SusDat), Physchem Data + Further Data (label 9, if users wish to provide additional physicochemical data), and Experimental Data (label 10, after querying *httk* and any available user-provided experimental data).

## 6.2 Data Collection

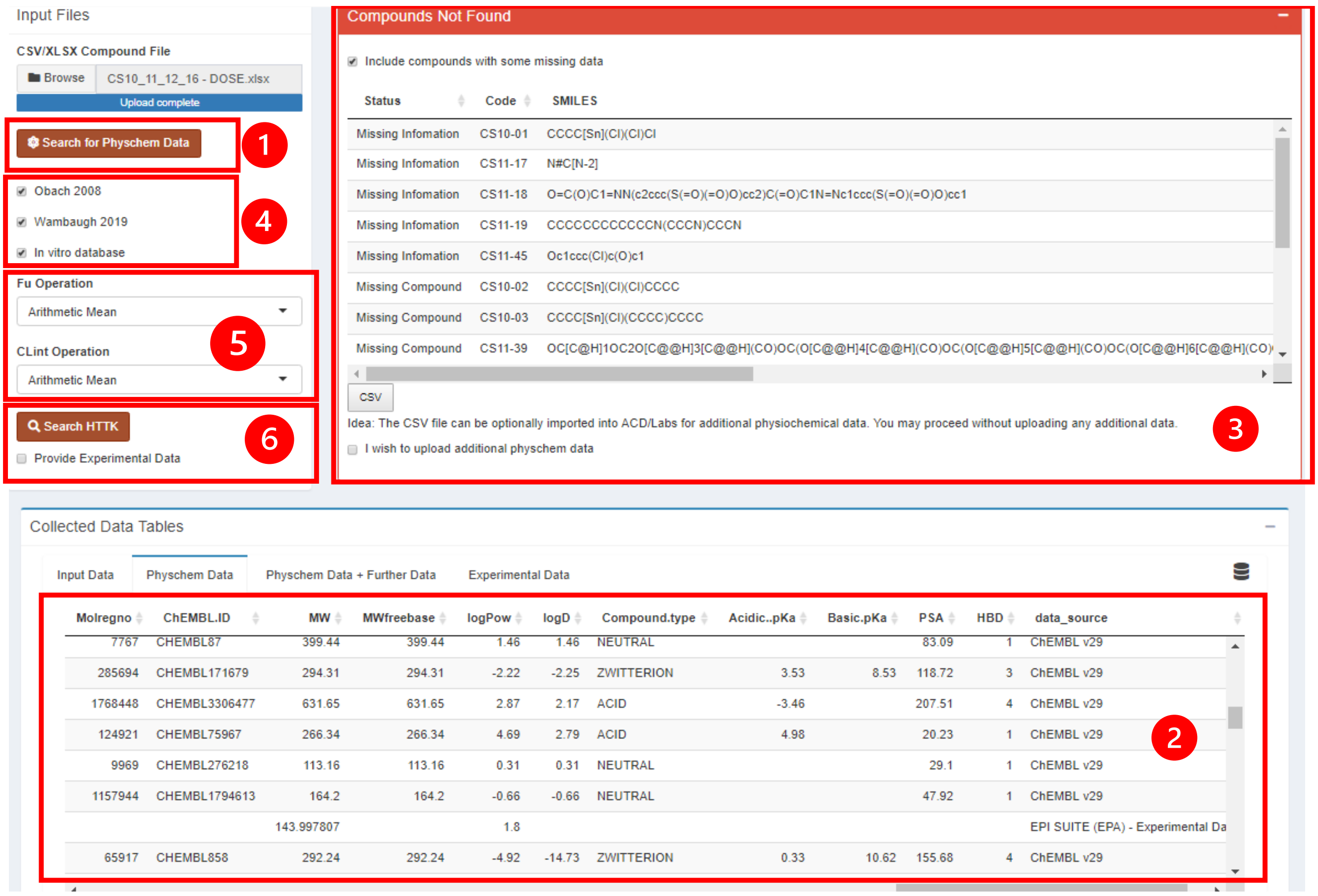


Figure S5: A Section of the Data Collection page of SimRFlow's R Shiny app. Upload of the Mandatory compound file allows the user to search for physicochemical data (button, label 1). After the search is complete, 3 additional elements appear on the screen: the collected physicochemical data (label 2), the compounds not found in the search (label 3), and prompts to proceed to the next step of experimental data collection (labels 4-6).

The Data Collection module begins by the upload of the Mandatory Compound file which is then processed. As shown in Figure S5, the upload of the mandatory compound file prompts the appearance of the 'Search Physchem data' button (label 1), which if clicked, returns all available physicochemical data on the compounds in the ChEMBL and SusDat databases. A table containing all of the collected physicochemical information appears under the 'Physchem Data' tab under the 'Collected Data Tables' box (label 2). Simultaneously, a box of missing compounds (or compounds with missing information) also appears, and prompts the user to optionally upload any available additional physicochemical data (label 3). If users choose to provide additional physicochemical data, it must be in the format outlined in the manuscript. The additional data is then automatically incorporated with the physicochemical data collected from ChEMBL and SusDat, and appears under the 'Physchem Data + Further Data' tab. If users wish to proceed to the next step of the data collection module (experimental data collection), they have the option to do so (label 4-6). To search through the 3 available *httk* databases, users must specify which of the 3 databases they wish to use (label 4), and which operation to apply on the collected $f_u$ and $CL_{int}$ data (label 5). Clicking the

'Search httk' (label 6) button will execute the search as per the user-specified inputs. Users may optionally upload PK-experimental data if they wish to do so (label 6).

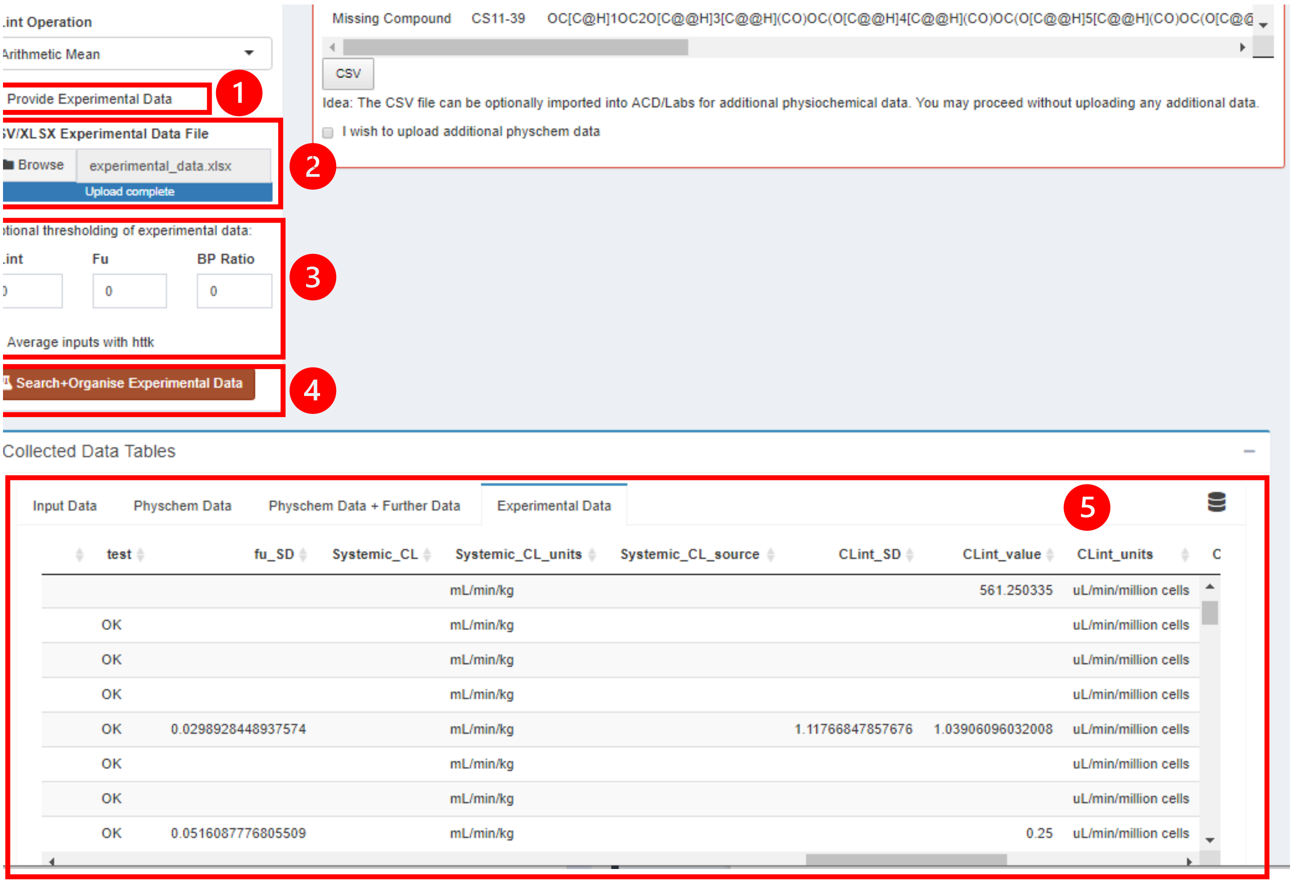


Figure S6: A Section of the Data Collection page of SimRFlow's R Shiny app. Upload of the optional experimental data file allows the integration of the user-provided experimental data with the collected physicochemical data form *httk*. The organised experimental and physicochemical data can be viewed and downloaded from the 'Experimental Data' tab.

The second phase of the data collection module is the search for experimental data in the *httk* databases, and optionally, the incorporation of user-provided experimental data. In cases where users do not have their own experimental data file, they must uncheck the box under label 1 in Figure S6. If the box is left unchecked, users will only search the *httk* databases for any available information on their compounds of interest (as shown in label 4 of Figure S5). If users can provide their own experimental data file, they must check the box under label 1 (Figure S6). This immediately prompts an upload bar to appear for the user to browse their device for the experimental data file (label 2). Once the experimental data file is uploaded, thresholding controls for $CL_{int}$, $f_u$ and BP ratio will immediately appear (label 3). If users do not wish to threshold their experimental data values, the default thresholds of 0 for all parameters will be used (values below 0 will be ignored). Finally, users may choose to either average their experimental data values with the data from *httk* by checking the 'Average with httk' checkbox (label 3). If the box is left unchecked, user-provided experimental data will be favoured over data from *httk*. Activating the experimental data search and organisation can be initiated by the search+organise button (label 4), which returns a table of all collected and organised physicochemical and experimental data under the 'Experimental Data' tab (label

5). The completion of the experimental data search completes all steps of the Data Collection module, and users can proceed to either the Simulation or Prediction modules.

## 6.3 Prediction

Clicking on the 'Prediction' option from the sidebar menu (label 1), takes the user to the prediction page (Figure S7). In order to use the prediction module, the user has to click the 'Predict Parameters' button (label 2) which initialises the Simcyp® v21 Engine which then predicts BP ratio, $f_u$, $V_{ss}$, and $K_d$ (for *HSA* or *AGP* depending on the compounds) using the collected physicochemical information. Any experimental data (form *httk* or from the user-provided file) will not be used for these predictions. The predictions are returned in a downloadable table (label 3).

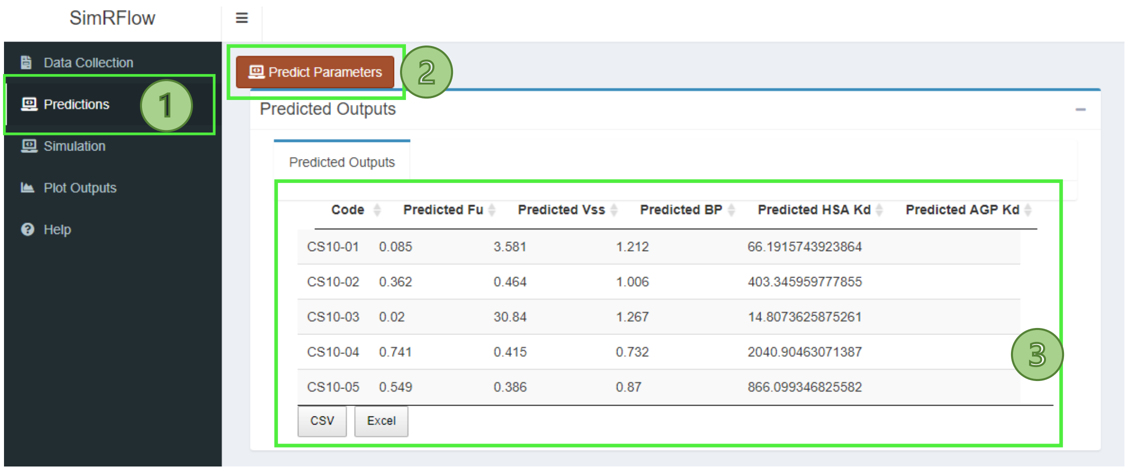


Figure S7: The Prediction module's page of SimRFlow's R Shiny app. Clicking the prediction button uses Simcyp® to predict BP ratio, $f_u$, $V_{ss}$ and $K_d$ value for the compounds, and returns the predictions as a downloadable table.

## 6.4 Simulation

Users can navigate to the Simulation module by selecting the 'Simulation' option from the side bar menu (label 1, Figure S8). Users can then select the $V_{ss}$ prediction method and the dose units for their simulation (label 2). In this example, users provided the dosing for each compound in their mandatory compound file, so the R Shiny app does not ask the user again to select the compound dosing for the simulations. If the user specified the $V_{ss}$ prediction method and the dose units in their mandatory compound file, they would not be prompted to specify them in the Simulation page. Users can also change the thresholds for the two main assumptions applied to the collected data (label 3). In this example, acidic compounds without an experimental BP ratio value are assumed to have a BP ratio of 0.55, and basic compounds with a pKa value that is greater than 7 are assumed to bind to *AGP* rather than *HSA*. Finally, users can design their simulations by setting the number of subjects, the simulation duration, and the route of administration form a drop down menu (label 4). Clicking the simulate button (label 5), initialises the Simcyp® v21 engine and runs compound-specific simulations using the collected physicochemical and experimental information as well as the user-specific simulation parameters. The simulated concentration-time profiles for all simulated subjects for each of the compounds appear under the 'Concentration-Time Profiles' tab (label 6), and the

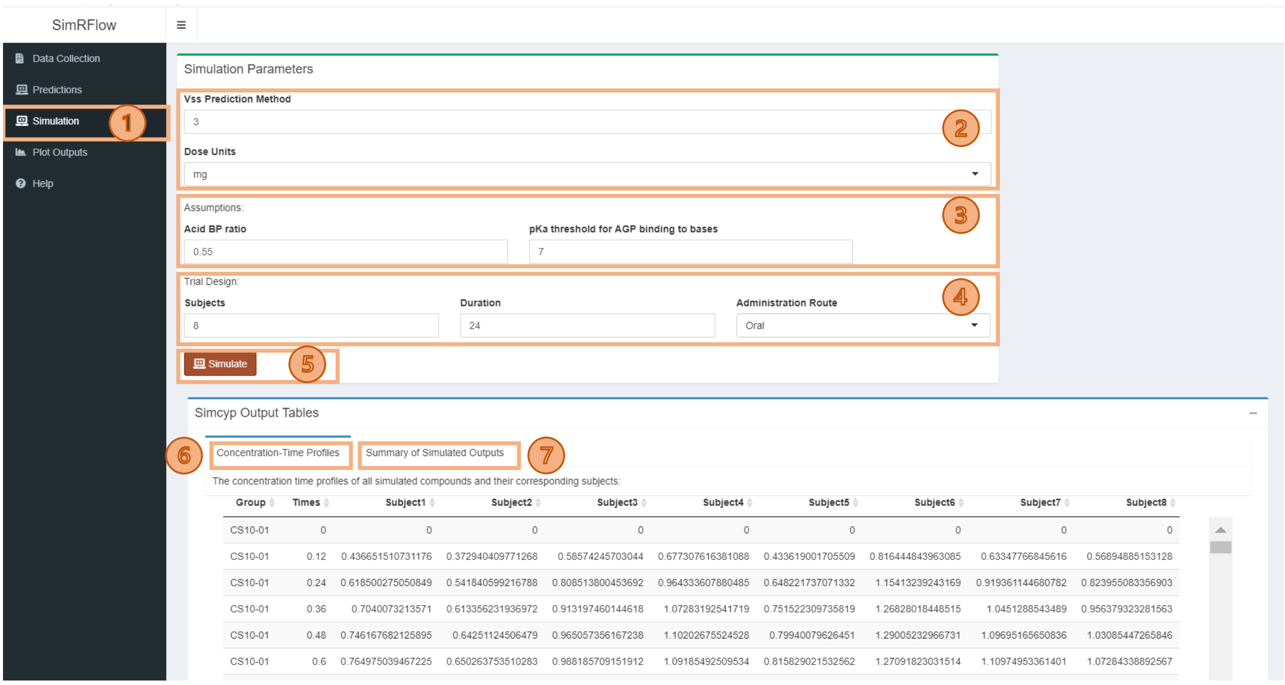


Figure S8: The Simulation module's page of SimRFlow's R Shiny app. Users can change multiple parameters before running the compound simulations and viewing the simulated parameters and profiles using the collected physicochemical and experimental data from the Data Collection module.

summary of additional outputs appear under the 'Summary of Simulated Outputs' tab (label 7). Both output tables are downloadable.

## 6.5 Data Visualisation

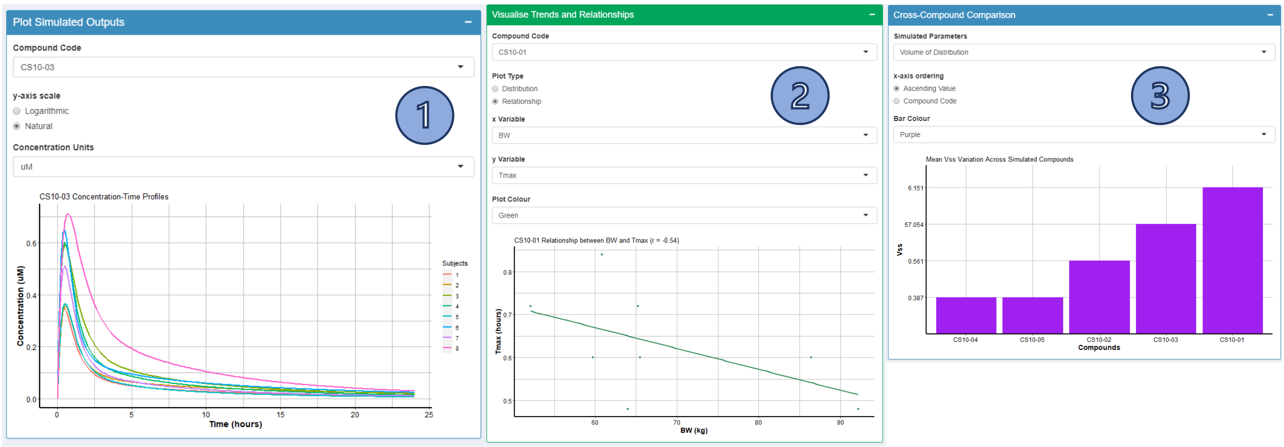


Figure S9: The Data Visualisation page of SimRFlow's R Shiny app. Three boxes are available simultaneously for plotting. Parameter distribution plots and relationship scatter plots can both be accessed by the 'Visualise Trends and Relationships' box.

For boxes labelled 1 and 2 in Figure S9, users can select the compound they wish to view from the drop-down menu titled 'Compound Code'. The 'Plot Simulated Outputs' box (label 1) allows the viewing of concentration-time profiles of all simulated subjects (up to 30 subjects). The concentration units can either be in $uM$ or in *ng/mL*, and the $y$-axis can either be in logarithmic or natural scales. The 'Visualise Trends and Relationships' box (label 2) provides the options of relationship scatter plots (where users should select the $x$ and $y$ axes parameters from a drop-down menu) and distribution plots (where users only select the x axis parameter from a drop-down menu). Plot colours can be changed from boxes labelled 2 and 3 from the drop-down menus titles 'Plot Colour' and 'Bar Colour', respectively. The 'Cross-Compound Comparison' box allows users to select a parameter from the drop-down menu titled 'Simulated Parameters'. The selected parameter average (across the simulated population) will be plotted for all simulated compounds. The bars can either be organised in ascending order (lowest parameter average to highest) or in compound-code order (alphanumeric ordering).

## REFERENCES

Chiba, M., Ishii, Y., and Sugiyama, Y. (2009). Prediction of hepatic clearance in human from in vitro data for successful drug development. *AAPS J* 11, 262–76. doi:10.1208/s12248-009-9103-6

Hinderling, P. H. (1997). Red blood cells: a neglected compartment in pharmacokinetics and pharmacodynamics. *Pharmacol Rev* 49, 279–95

Watanabe, R., Esaki, T., Kawashima, H., Natsume-Kitatani, Y., Nagao, C., Ohashi, R., et al. (2018). Predicting fraction unbound in human plasma from chemical structure: Improved accuracy in the low value ranges. *Mol Pharm* 15, 5302–5311. doi:10.1021/acs.molpharmaceut.8b00785
